# Design, Synthesis, and In Vitro Evaluation of Novel 8-Amino-Quinoline Combined with Natural Antioxidant Acids

**DOI:** 10.3390/ph15060688

**Published:** 2022-05-31

**Authors:** Andrea Bacci, Francesca Corsi, Massimiliano Runfola, Simona Sestito, Ilaria Piano, Clementina Manera, Giuseppe Saccomanni, Claudia Gargini, Simona Rapposelli

**Affiliations:** 1 Department of Pharmacy, University of Pisa, Via Bonanno 6, 56126 Pisa, Italy; andrea.bacci@phd.unipi.it (A.B.); francesca.corsi@phd.unipi.it (F.C.); massimiliano.runfola@farm.unipi.it (M.R.); ilaria.piano@unipi.it (I.P.); clementina.manera@unipi.it (C.M.); giuseppe.saccomanni@unipi.it (G.S.); maria.gargini@unipi.it (C.G.); 2 Department of Chemistry and Pharmacy, University of Sassari, Via Vienna 2, 07100 Sassari, Italy; ssestito@uniss.it; 3 Center for Instrument Sharing (CISUP), University of Pisa, 56126 Pisa, Italy

**Keywords:** antioxidant, natural products, neurodegenerative diseases, oxidative stress, multitarget, retinitis pigmentosa, drug discovery

## Abstract

Overproduction of reactive oxygen species (ROS) and alterations in metallostasis are common and related hallmarks in several neurodegenerative diseases (NDDs). Nature-based derivatives always represent an attractive tool in MTDL drug design, especially against ROS in NDDs. On this notion, we designed a new series of 8-quinoline-N-substituted derivatives with a natural antioxidant portion (i.e., lipoic, caffeic, and ferulic acids). These compounds were shown to chelate copper, a metal involved in ROS-induced degeneration, and scavenger oxygen radicals in DPPH assay. Then, selected compounds **4** and **5** were evaluated in an in vitro model of oxidative stress and shown to possess cytoprotective effects in 661W photoreceptor-like cells. The obtained results may represent a starting point for the application of the proposed class of compounds in retinal neurodegenerative diseases such as retinitis pigmentosa (RP), comprising a group of hereditary rod–cone dystrophies that represent a major cause of blindness in patients of working age, where the progression of the disease is a multifactorial event, with oxidative stress contributing predominantly.

## 1. Introduction

Oxidative stress (OS) is a complex network of malfunctioning pathways resulting from an imbalance of oxidant and antioxidant processes occurring within cells and tissues. Balanced levels of reactive oxygen species (ROS) and nitrogen species (RNS) are deputed to physiological processes and are finely regulated for cell differentiation and migration, immune response, and apoptosis [1]. When this equilibrium is altered, ROS induce a progressive loss of tissues’ function, thus contributing to the onset and/or progression of several neurodegenerative disorders [2]. Despite heterogeneity, OS represents a common hallmark in the pathogenesis of uncurable neurodegenerations including Parkinson (PD), Alzheimer (AD), Huntington (HD), Batten (BD) diseases, and amyotrophic lateral sclerosis (ALS) [3,4,5]. In this context, retinal neurodegenerations are a group of different disorders associated with retinal tissue. For example, age-related macular degeneration (AMD) is a multifactorial disease in which OS drives a series of events such as protein misfolding and deposition. In addition, retinitis pigmentosa (RP) is an orphan genetic disease (ORPHA:791) in which the primary death of rods carrying the mutation causes an increase in retinal oxygen levels. This event triggers the increment in ROS production and supports the progression of the disease, leading to secondary neurodegeneration of the cones. Several studies also reported that antioxidant molecules are effective in slowing cone death in in vivo models of RP with different genetic mutations, indicating OS as the starting factor of cone degeneration [6,7,8,9]. At the same time, OS is involved in several other disorders not strictly related to neurodegeneration, including cellular aging, cancer, and cardiovascular, diabetes, and autoimmune disorders [10,11].

**Figure 1:**
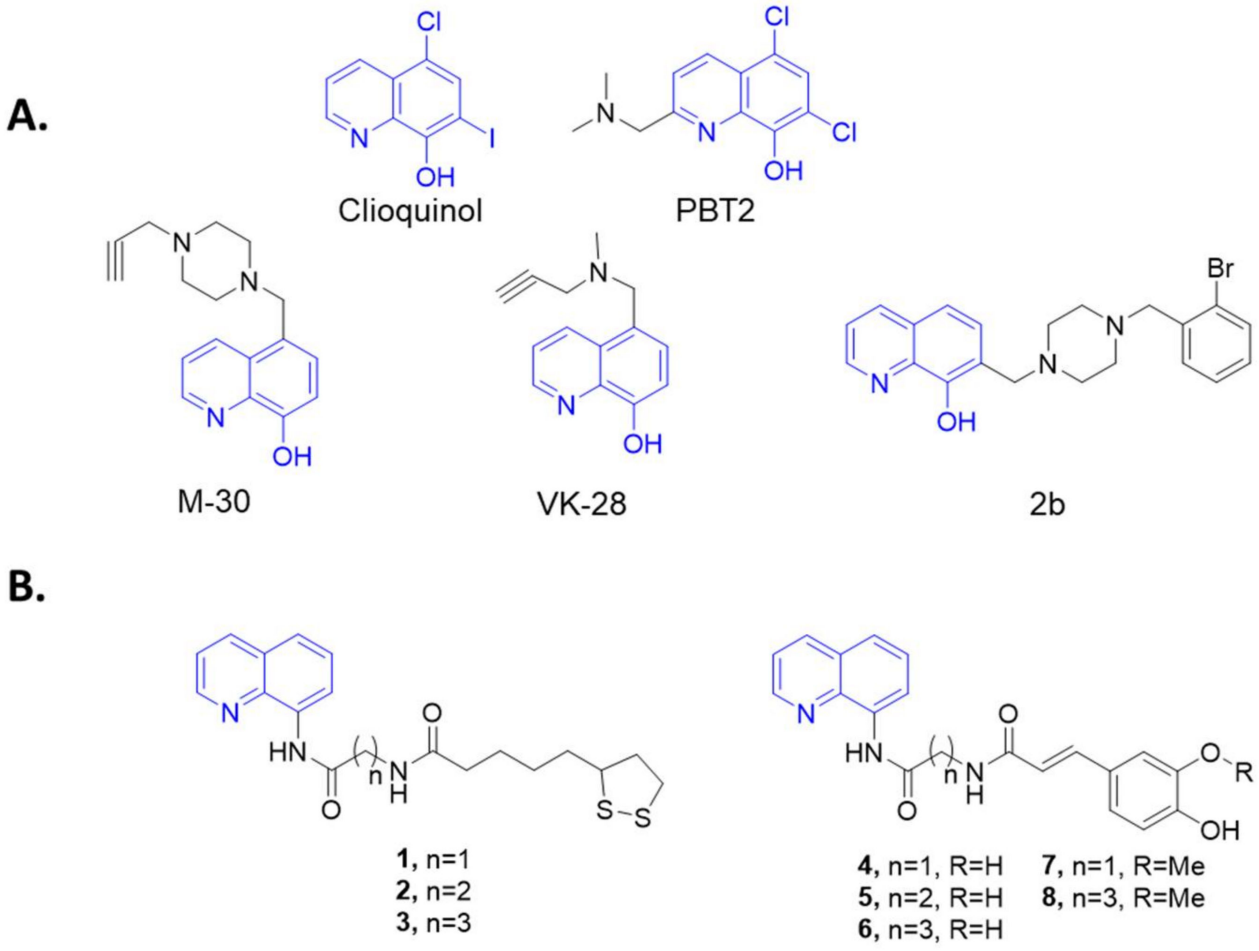
(**A**) Structures of M-30, VK-28, and 2b; (**B**) General structure of designed compounds merging 8-aminoquinoline scaffold with natural acids are reported in the box.

ROS could originate from exogenous sources including exposure to irradiations and toxic substances, but endogenous mechanisms (e.g., mitochondria respiratory chain) are the most predominant. Mitochondrial ROS are produced by the electron transfer chain (ETC), a series of protein complexes located in the mitochondrial membrane [12]. ROS accumulation is harmful to cellular homeostasis and actively contributes to a vicious circle that leads to an uncontrolled inflammatory status, proteins’ misfolding and aggregation, lipid peroxidation, and the overactivation of innate immune defense players, such as microglia and astrocytes [13]. Along with OS, metallostasis represents another key factor both in the insurgence and progression of cellular damage and contributes to destabilizing the conformation of biostructures including lipids, DNA, and proteins [14,15]. Although OS and inflammation are well-known features of neurodegenerative diseases, recent studies report oxidative damage as one of the main causes of cones’ death in RP [16,17]. This laid the foundation to investigate the protective role of phytonutrients in retinal diseases such as RP and diabetic retinopathy [18,19]. In particular, nutraceuticals including *N*-acetylcysteine (NAC) [20], sulforaphane [21], naringenin, and quercetin have been proven to delay retinal degeneration in preclinical models of RP [6]. Within the arsenal of compounds tested, NAC showed to be particularly effective in promoting the cone survival and function in a mouse model of RP by supporting its transition from preclinical to clinical studies (phase I, clinicaltrial.gov NCT03063021) [22] designed to define its safety and tolerability in patients with RP. In the last decades, metal chelators such as clioquinol and PBT2 (Figure 1) entered clinical trials in several neurodegenerative diseases including AD, PD, and HD. Unfortunately, they have been stopped because of the induction of adverse effects or because they failed to meet the clinical end points [23]. In order to design novel compounds with a wider spectrum of activities for the treatment of AD, PD, and HD, a plethora of multi-directed target ligands (MTDLs) through the combination of metal-chelating agents and frameworks with antioxidant and neuroprotective activities have been synthetized (M-30, VK-28, HLA-20) [24,25,26]. Indeed, this approach is currently pursued for the development of disease-modifying drugs for the treatment of neurodegenerative [27,28,29,30] and orphan diseases [31,32], which still lack effective therapies (Figure 1).

Taking this into account, a new series of 8-quinoline-N-substituted derivatives **1**–**8** have been designed and synthetized by linking the 8-aminoquinoline scaffold, as chelating scaffold, with natural-based antioxidant compounds including ferulic (FA), caffeic (CA), and lipoic (LA) acids [33] (Figure 1), which have been widely investigated for their antioxidant behavior and have been proven to directly neutralize free radicals [34,35,36,37,38]. Compounds **1**–**8** were tested for their ROS-scavenging and copper-chelating properties. Finally, an in vitro photoreceptor-like model (661W cells) of H_2_O_2_-induced oxidative damage was used to evaluate cytoprotective and antioxidant activity by assessing cell ROS+ and levels of acrolein produced, a biomarker of oxidative stress.

## 2. Results

### 2.1. Chemistry

**Scheme 1:**
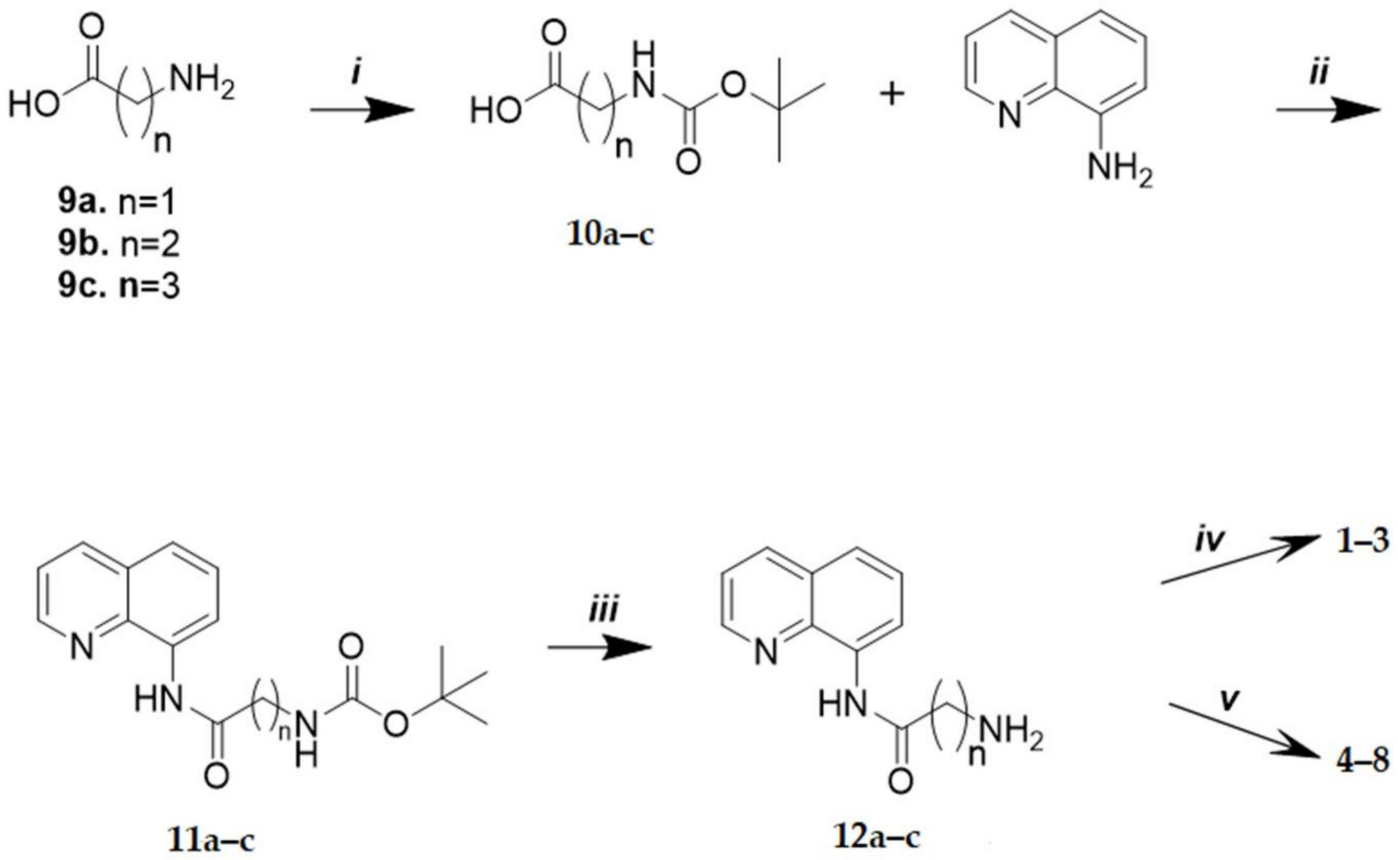
Reagents and conditions: **(i**) Boc_2_O, NaOH (1M)/i-PrOH (4:3), rt, 2 h; (**ii**) TBTU, DIPEA, anhydrous DMF, N_2_, rt, 12 h; (**iii**) TFA, DCM, −10/−20 °C, 3 h; (**iv**) lipoic acid, TBTU, DIPEA, anhydrous DMF, N_2_, rt, 12 h; (**v**) caffeic or ferulic acid, DCC, DMAP, DCM, DMF, rt, 12 h.

Compounds **1**–**8** were synthetized, as depicted in Scheme 1. Briefly, protected amines **10a–c** were obtained by the reaction between the amino acid **9a**–**c** (i.e., glycine, β-alanine, or γ-aminobutyric acid) and di-tert-butyl dicarbonate. Then, the condensation of **10a**–**c** with 8-aminoquinoline provided the amides **11a**–**c**. N-Boc adducts **11a–c** were then deprotected with TFA, affording the free amines **12a**–**c,** which were condensed with lipoic, caffeic, and ferulic acids, yielding the final products **1**–**8**. The amide-coupling reaction with lipoic acid and **12a**–**c** was performed with TBTU/DIPEA as coupling reagents, whereas the same reaction with caffeic and ferulic acids was performed replacing TBTU/DIPEA with DCC/DMAP.

### 2.2. Copper-Chelating Activity Test

**Figure 2:**
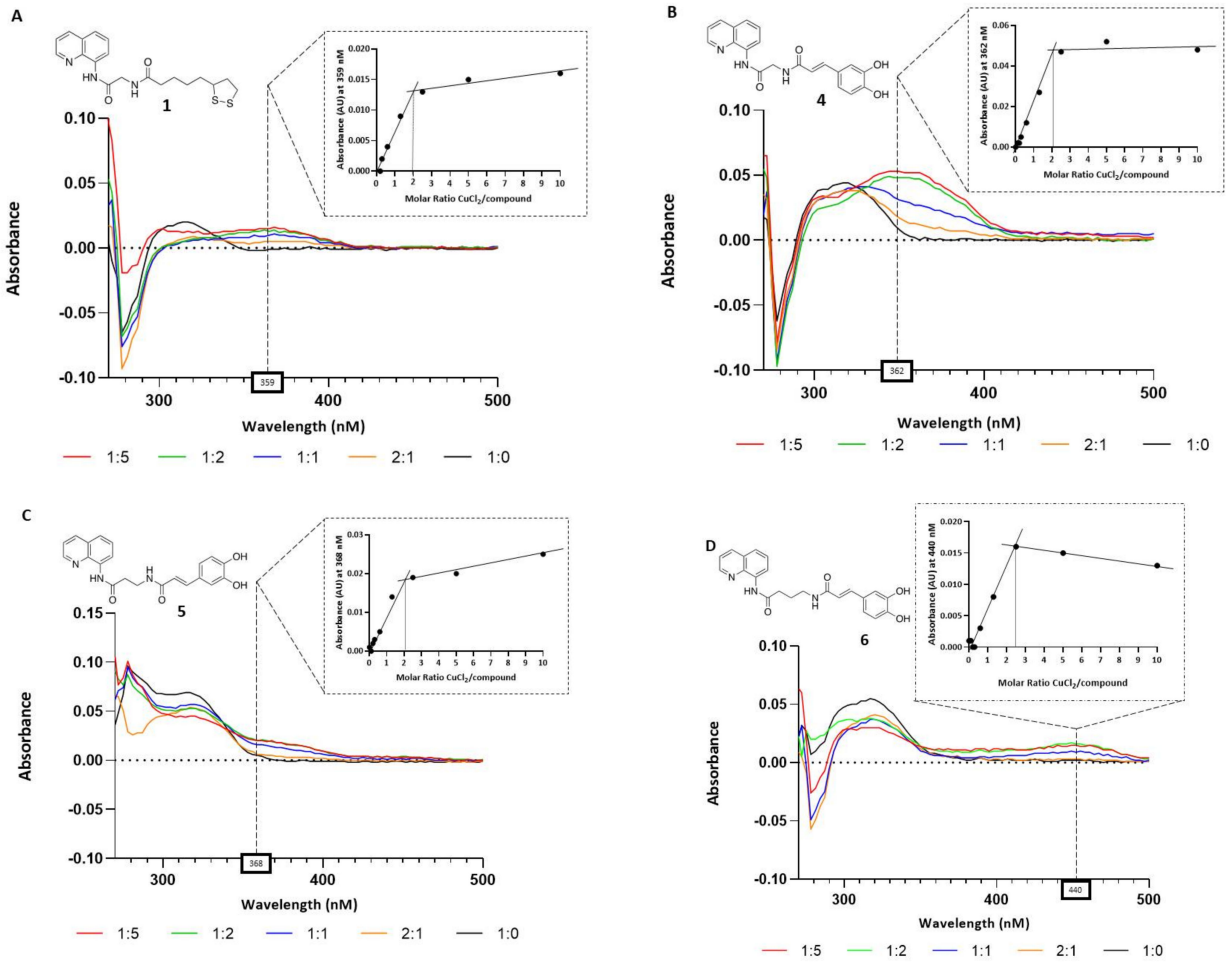

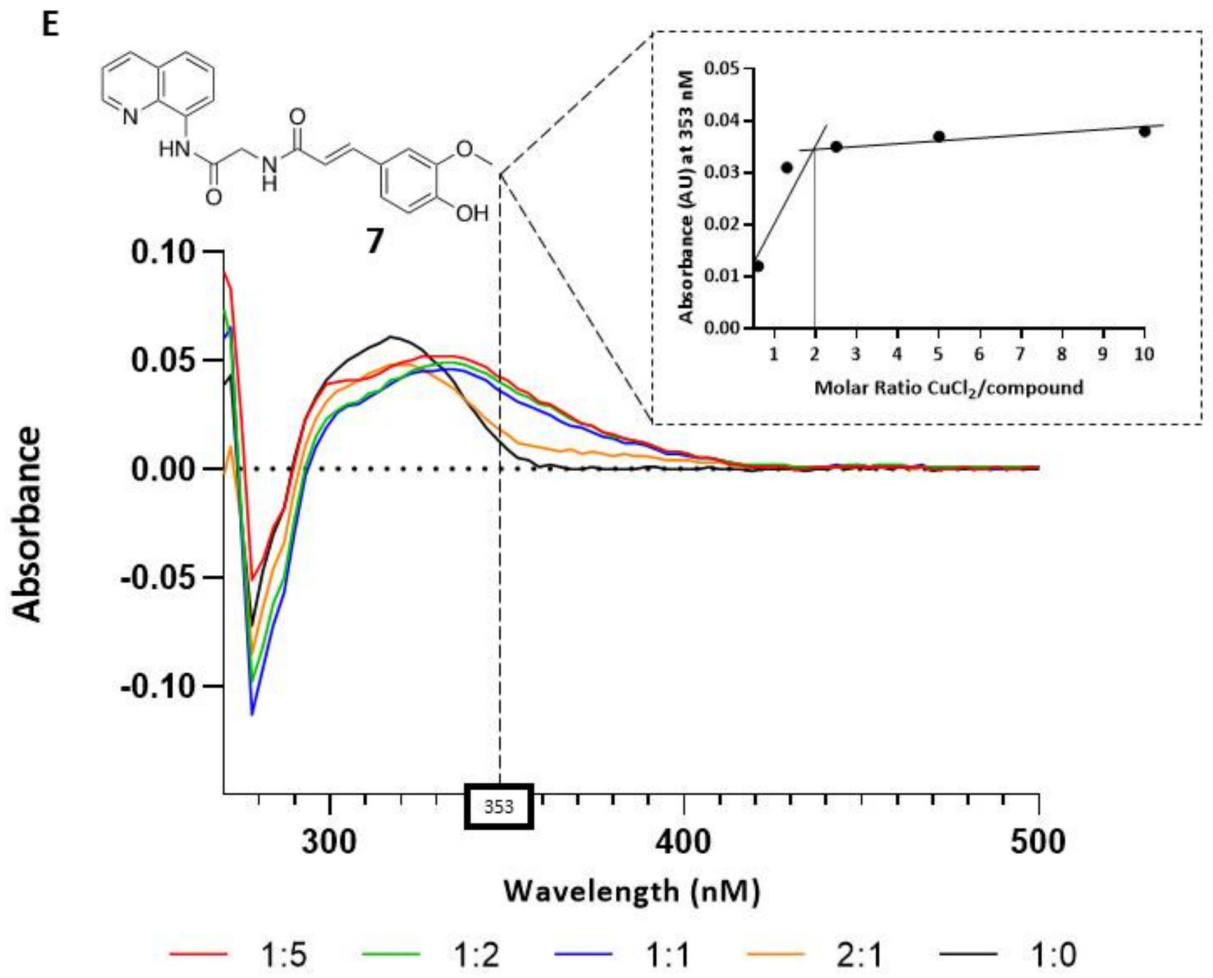
Copper-chelating activity of compounds **1**, **4**, **5**, **6**, and **7**. Graphs (**A**–**E**): UV spectra of the compounds alone (10 μM, in black line) and treated with increasing concentrations of CuCl_2_ (5, 10, 20, and 50 μM). The box on the top of each graph shows the Cu^2+^-ligand complex molar ratio at the maximum variation of absorbances for the tested compounds (359 nm for **1**, 362 nm for **4**, 368 nm for **5**, 440 nm for **6**, 353 nm for **7**).

Compounds **1**–**8** were preliminarily tested for their capacity to chelate copper ions by UV-Vis spectroscopy. The specific absorbance peaks of compounds **1–8** were monitored in the range of 270–500 nm (Figure 2). The co-incubation of 10 µM of the tested compound together with increasing amounts of CuCl_2_ induced an isosbestic shift of the absorption for compounds **1**, **4**, **5**, **6,** and **7,** indicating the formation of Cu^2+^–compound complexes. On the contrary, derivatives **2**, **3**, and **8** were found to be devoid of chelating activity (data not shown). We also evaluated the molar ratio between chelating ligands and copper ions. The stoichiometry of the Cu^2+^–compound complex was determined by using the molar ratio method (Figure 2). The maximum variations of absorbance were recorded with a 10 μM solution of the tested compounds and an increased concentration of CuCl_2_ (0–50 μM). As shown, the absorbance initially increased until a breakpoint, and the intersection of the two straight lines indicates the mole fraction of Cu^2+^–ligand complexes. From the analyzed data, we obtained that lipoic derivative **1**, caffeic compounds **4** and **5,** and ferulic ligand **7** gave a mole fraction of 2, indicating a 2:1 stoichiometry for the Cu^2+^–ligand complex, whereas the Cu^2+^–**6** complex showed a 2.5:1 stoichiometry.

### 2.3. Radical-Scavenging Activity

**Figure 3:**
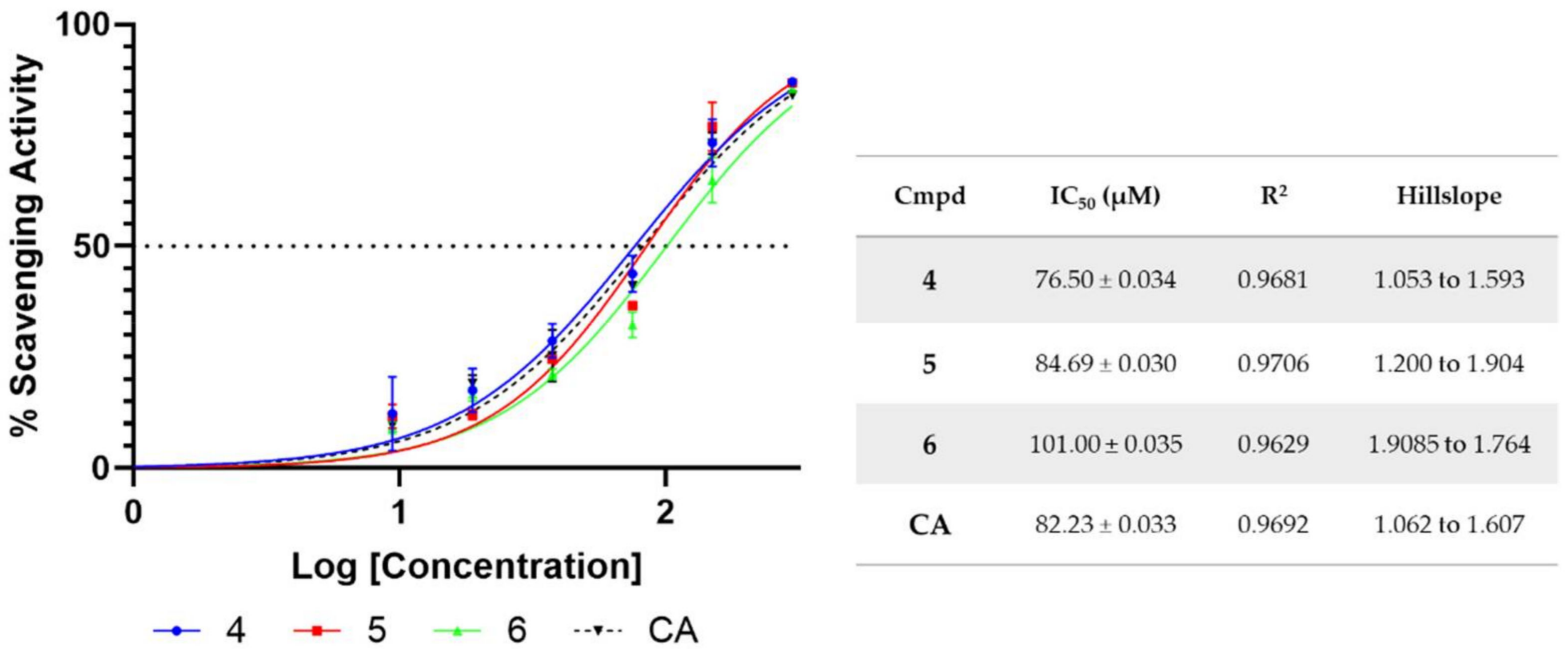
Dose–response curve of DPPH radical-scavenging activity for compounds **4**–**6** and caffeic acid. Compounds were incubated for 45 min at descending concentrations (300–0 μM) with 500 μM DPPH solution. IC_50_ values ± SD are indicated in the table. Data are from duplicates.

The radical-scavenging properties of derivatives **1**–**8** were evaluated using a spectrophotometric in vitro assay with 1,1-diphenyl-2-picryl-hydrazyl (DPPH). Compounds **1–8** and their relative phytonutrient precursors (LA, CA, and FA) were initially tested at three different concentrations (200, 100, and 50 µM) and incubated for 45 min with a 500 µM DPPH methanolic solution. The antioxidant activity is depicted in Table 1 as the mean values ± standard deviations. Caffeic derivatives **4**–**6** were good antioxidants, showing a percentage of radical scavenging higher than caffeic acid (63.9%). Hence, the IC_50_s (defined as the concentration resulting in a 50% scavenging activity) were determined (Figure 3). Although the FA derivatives showed the same scavenging capacity as the parent compound (e.g., FA) at all concentrations tested, the lipoic acid derivative **2** possessed only a modest scavenging capacity, at 200 µM, whereas **1** and **3** proved to be devoid of antioxidant activity.

### 2.4. Biological Evaluation of Compound in H_2_O_2_-Induced Cell Death

**Figure 4:**
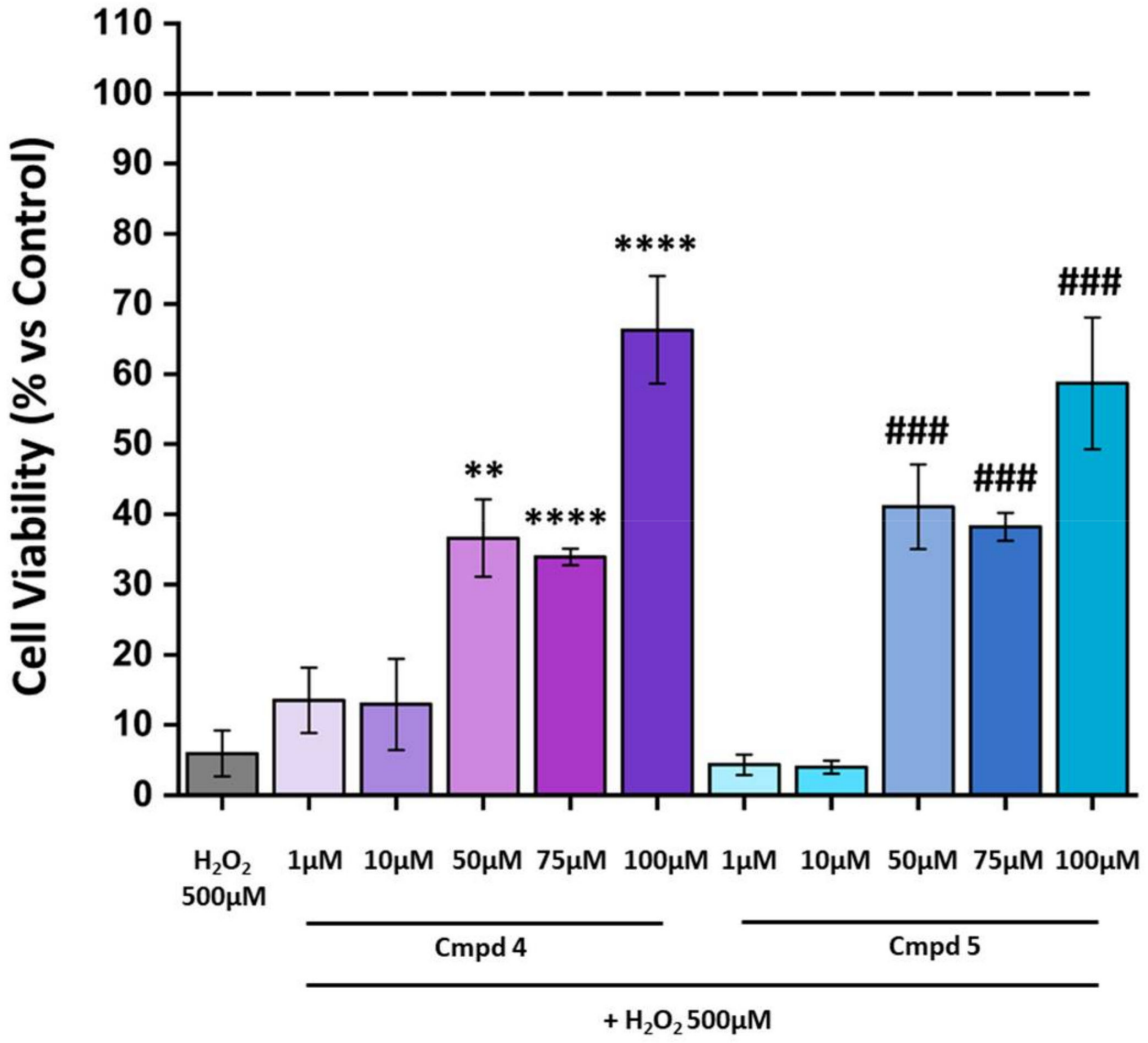
Treatment with **4** or **5** at 100 µM decreases cell death due to damage with H_2_O_2_. Cell viability was analyzed by CellTiter 96 AQueous One Solution Reagent. The cells were pre-treated for 24 h with compounds **4** or **5** at various concentrations (1–100 µM) and then exposed for 3 h with H_2_O_2_ 500 µM. The dashed line indicates the reference value of the Ctrl (control group: no compounds or H_2_O_2_ exposure). The values in the graph indicate % viability as the mean ± SE obtained from *n* = 3 of independent experiments. Statistics: one-way ANOVA test followed by Levene’s post-test; ** *p* ≤ 0.01 (**4**: 50 µM vs. H_2_O_2_ 500 µM); **** *p* ≤ 0.0001 (**4**: 75 µM vs. H_2_O_2_ 500 µM; **4**: 100 µM vs. H_2_O_2_ 500 µM); ### *p* ≤ 0.001 (**5**: 50 µM vs. H_2_O_2_ 500 µM; **5**: 75 µM vs. H_2_O_2_ 500 µM; **5**: 100 µM vs. H_2_O_2_ 500 µM).

To further evaluate whether compounds **1**–**8** protect against OS-induced cell death, we assessed their efficacy in preserving the H_2_O_2_-induced 661W cell line death at five different concentrations (1–100 µM). The range of concentrations was chosen according to the antioxidant activity of the compounds observed through the DPPH assay. The administration of H_2_O_2_ (500 µM for 3 h) to the cells led to an approximately 90% decrease in cell viability. All compounds tested were unable to protect the cells (see Figure S1 in Supporting Materials) except for compounds **4** and **5,** which induced a significant reduction in H_2_O_2_-induced damage, preserving cell viability (Figure 4). Based on the results, despite an increase in cell viability being found at concentrations of 50 and 75 µM, after the H_2_O_2_-induced cell death, 100 µM was considered the most suitable concentration of **4** and **5** to be used for further experiments.

### 2.5. Compounds **4** and **5** Protect 661W Photoreceptors from H_2_O_2_-Induced Cell Death by Reducing Intracellular ROS Generation

**Figure 5:**
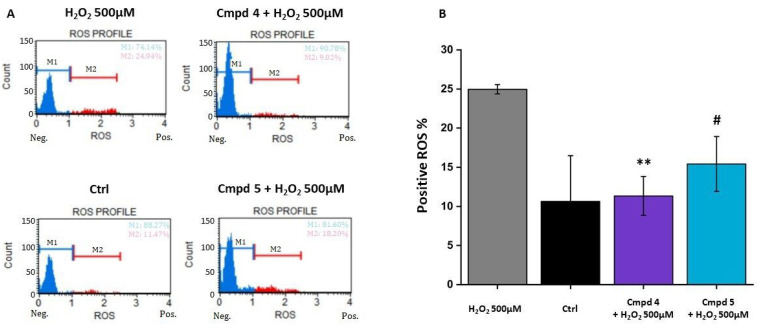
**Treatment with 4 or 5 reduces the production of reactive oxygen species (ROS).** Cells were pre-treated for 24 h with compounds **4** or **5** at the chosen concentration of 100 µM and then exposed for 3 h with H_2_O_2_ 500 µM. (**A**) Representative histograms of ROS-negative (M1, blue) and ROS-positive (M2, red) cells; (**B**) The bar graph shows the percentage of ROS-positive cells. (Ctrl: control group, no compounds or H_2_O_2_ exposure). Values in the graph indicate % of ROS+ cells as the mean ± SE obtained from *n* = 3 of independent experiments; statistics: one-way ANOVA test followed by Levene’s post-test; ** *p* ≤ 0.01 (**4**: 100 µM vs. H_2_O_2_ 500 µM; # *p* ≤ 0.05 (**5**: 100 µM vs. H_2_O_2_ 500 µM).

**Figure 6:**
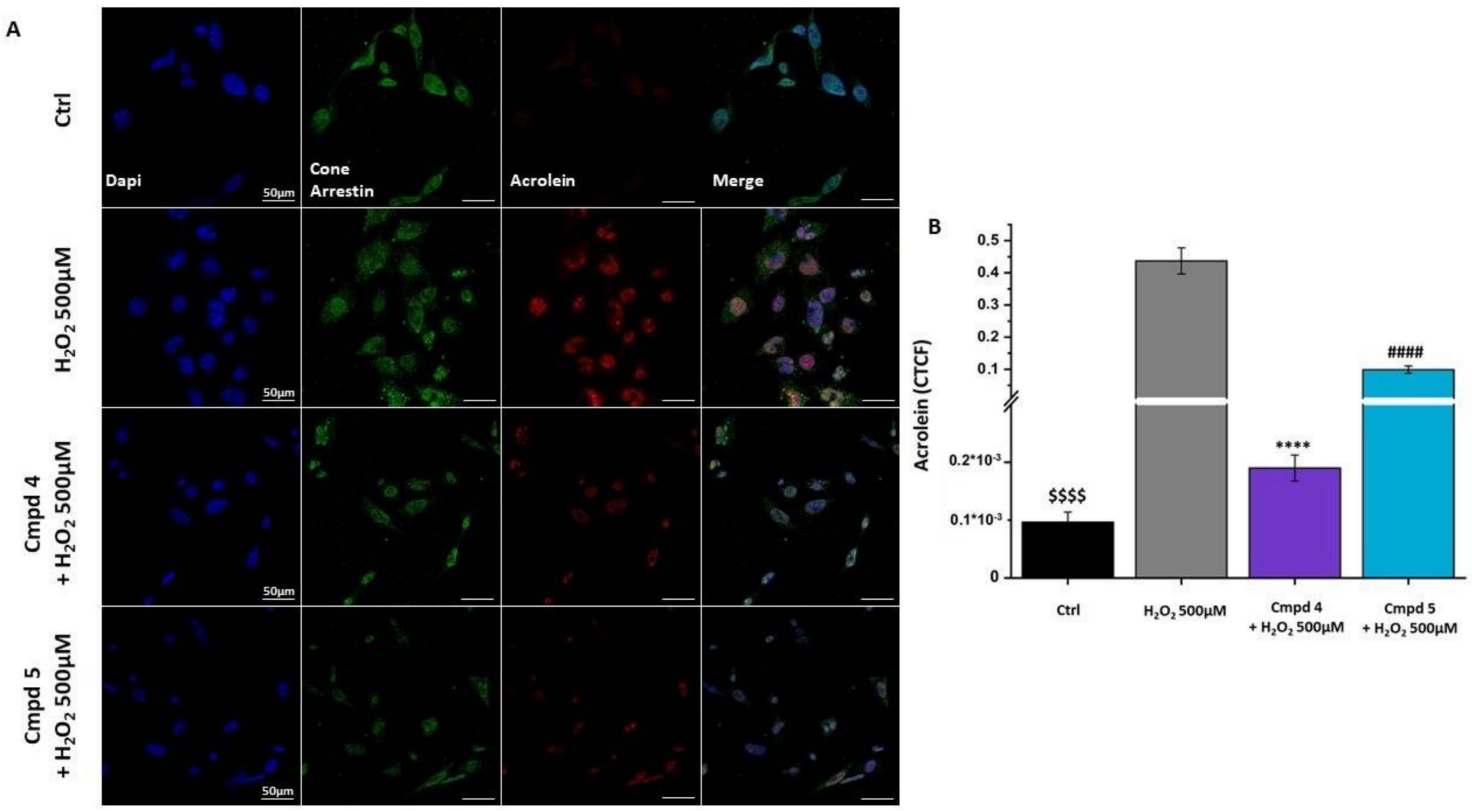
**Treatment with 4 or 5 decreases the expression of oxidative stress biomarker in 661W cell line.** (**A**) Immunofluorescence detection of cone arrestin (green) and acrolein (red) in cells pre-treated with compounds **4** or **5** for 24 h at the chosen concentration of 100 µM and then exposed for 3 h to H_2_O_2_ 500 µM. Nuclei were counterstained with DAPI. (Ctrl: control group, no compounds or H_2_O_2_ exposure). Scale bars = 50 µm; (**B**) Corrected total cell fluorescence (CTCF) of acrolein in cell treated as indicated. Values in the graph indicate the CTCF of acrolein of cells as the mean ± SE, normalized with the H_2_O_2_ 500 µM group (see Materials and Methods section for CTCF calculation); statistics: one-way ANOVA test followed by Levene’s post-test. $$$$ *p* ≤ 0.0001 (Ctrl vs. H_2_O_2_ 500 µM); **** *p* ≤ 0.0001 (**4**: 100 µM vs. H_2_O_2_ 500 µM); #### *p* ≤ 0.0001 (**5**: 100 µM vs. H_2_O_2_ 500 µM).

We then verified whether derivatives **4** and **5** attenuate H_2_O_2_-mediated cell death by suppressing intracellular ROS generation. Flow cytometry data showed that treatment with H_2_O_2_ resulted in an increase in intracellular ROS generation in the 661W cell line compared to the control group (Figure 5A). The bar graph in Figure 5B shows the percentage of the ROS-positive (ROS+) cell population in the control (black bar), stressed cells (gray bar), and the cells pre-treated with the selected compounds **4** and **5** (purple and light-blue bar, respectively). From the graph, it is possible to observe that the pre-treatment was able to significantly reduce the percentage of ROS+ cells. To validate the protective function of the two compounds against OS, we assessed the expression of acrolein, which is a marker of lipid peroxidation induced by oxidative stress. In the panel in Figure 6A, representative images, acquired by confocal microscopy, showing the presence of acrolein adducts (red staining) following damage with H_2_O_2_ in the 661W cells, in green show a cell-specific marker (cone arrestin) and, in blue, the nuclei. The results obtained by the acquisition of the confocal image were confirmed by the quantification of acrolein cell fluorescence (CTCF), summarized in Figure 6B. Acrolein expression increased considerably in the 661W cells after exposure to H_2_O_2_; however, pre-treatment with **4** or **5** seemed to significantly reduce the intensity of fluorescence relative to the acrolein, validating the antioxidant effect of the tested molecules. Overall, these results suggested that **4** and **5** reduce the H_2_O_2_-induced intracellular ROS generation in the 661W photoreceptor-like cells, leading to a decrease in oxidative stress-mediated cell death.

## 3. Discussion

In our previous studies, we demonstrated that the introduction of phytonutrients including lipoic, caffeic, and ferulic acids into the chemical scaffold of acetylcholinesterase inhibitors such as rivastigmine [32] and tacrine [33,34,35] leads to new molecules, which retain the cholinesterase inhibitory activity and show antioxidant and chelating properties, thus proving to be valuable disease-modifying candidates to treat AD. Following the same strategy and aiming at identifying new compounds with good antioxidant and metal-chelating profiles, herein we described the synthesis and the preliminary evaluation of a small series of new compounds obtained by linking LA, FA, and CA with the 8-amminoquinoline scaffold, known to possess chelating properties [36,37]. The choice of nature-based acids relies on the solid knowledge of their safe profiles and, moreover, on additional effects beyond their direct antioxidant activity. The activation of antioxidant enzymes such as SOD (superoxide dismutase) or CAT (catalase), antioxidant proteins such as GSH (glutathione), and autophagic machinery stimulation represents attractive aspects that support their use in anti-AD drug discovery [39,40,41]. The novel 8-amminoquinoline derivatives were then evaluated for their capability to chelate copper by UV-Vis spectroscopy. Results indicated that five out eight compounds were able to complex Cu^2+^ with a stoichiometry of 2:1, whereas lipoic acid derivatives **2** and **3** were unable to chelate Cu^2+^. Further, we performed a DPPH assay to evaluate the radical-scavenging ability. As expected, the newly synthetized molecules retained the scavenging properties of the parent compounds, at least at higher concentrations. Of note, derivatives **4**–**6** showed the best antiradical activity, while **7**–**8** resulted to be two-thirds fold less active than the caffeic analogues (**4**–**6**), indicating the importance of the catecholic portion for the antioxidant potential. In order to evaluate the new molecules as potential tools to fight OS, we assessed the cytoprotective effects in the H_2_O_2_-induced damage of a photoreceptor-like cell line. Photoreceptors are prone to oxidative stress, mainly from a high metabolic rate, and OS alteration may lead to retinal dysfunction and progressive neurodegeneration of cone photoreceptors, which can be worsened by the disruption of metal ion homeostasis. Considering the key role of both ROS and metal ions in OS-induced damage, compounds **1**–**8** were assessed for protection against oxidative stress. Within the compounds synthetized, **4** and **5** proved to protect the 661W photoreceptors from H_2_O_2_-induced cell death by reducing intracellular ROS generation at concentrations in the range of 50–100 μM. Moreover, both compounds were able to decrease the expression of acrolein, a marker of lipid peroxidation induced by oxidative stress. Additionally, further investigation will be needed to elucidate whether the cytoprotective effect induced by compounds 4 and 5 is essentially due to the scavenger and chelating properties or is the result of a more complex multifunctional activity due to a polypharmacological profile.

## 4. Materials and Methods

### 4.1. Synthesis and Characterization

General Material and Methods. Commercial-grade anhydrous solvents were used without further drying. Commercially available chemicals were purchased from Sigma-Aldrich, Fluorochem, or Alfa Aesar and used without further purification. Evaporation was performed in a vacuum (rotating evaporator). Anhydrous Na_2_SO_4_ was always used as the drying agent. Flash chromatography was performed on Merck 60 Å high-purity grade silica gel (0.40–63 μm). Reactions were followed by TLC, performed on Merck aluminum silica gel (60 F254) sheets. Spots were viewed under a UV lamp (254 nm) or with the aid of 10% phosphomolybdic acid in EtOH. Celite^®^ 545 was used as a filter agent. All melting points were determined on a Barnstead/Electrothermal MeL-Temp Model 1101D and were uncorrected. The ^1^H and ^13^C NMR spectra were obtained using a Bruker TopSpin 3.2 400 MHz Spectrometer and were recorded at 400 and 101, respectively. Chemical shifts are reported in parts per million (ppm) δ values, downfield from the internal reference tetramethylsilane (TMS), and referenced from solvent resonance as the internal standard: deuterochloroform (δ 7.26 (1H spectra), δ 77.16 (13C spectra)); deuterodimethylsulfoxide (δ 2.50 (1H spectra), δ 39.52 (13C spectra)); deuteromethanol (δ 3.31 (1H spectra)). Coupling constants J are reported in Hertz (Hz). The ^13^C NMR spectra were ^1^H decoupled. Signal patterns are indicated as follows: singlet (s), doublet (d), triplet (t), double-doublet (dd), double-triplet (dt), multiplet (m), broad singlet (br s), broad doublet (br d), broad triplet (br t), and broad multiplet (br m). High-resolution mass spectra were recorded on a ABSciex 3200 QTRAP using electrospray positive ionization. HPLC purity determination was performed on a Varian Pro Star 330PDA detector, a ternary HPLC pump Varian 9012, and a Rheodyne injector with 20 μL loop. An RP column ThermoScientific TM Hypersil TM C18 ODS (5 μm, 250 × 4.6 mmID) HPLC was used for all analyses (detection at 240 nm). Stock solutions of compounds **1**–**8** were prepared in a mixture of MeOH/ACN (2:3) and stored at 4 °C. The mobile phase was acetonitrile/water, and the elution gradient varied, according to the method depicted in the Supplementary section. HPLC analysis confirmed the ≥95% purity of all compounds, **1**–**8**.

#### General Procedure for the Synthesis of Compounds **1**–**3**

To a solution of commercial lipoic acid (30 mg, 0.14 mmol), in dry DMF, under N_2_ atmosphere, and cooled to 0 °C, TBTU (46 mg, 0.14 mmol) and DIPEA (0.05 mL, 0.29 mmol) were added. After 30 min at 0 °C, the intermediate **12a**–**c** (0.14 mmol) was added, and the temperature was kept at 0 °C for an additional 30 min. Later, the mixture was slowly warmed to rt and left under stirring overnight. Then, the organic solvent was reduced under vacuum, and the crude mixture was treated with H_2_O. The resulting precipitate was collected by filtration, dried, and further purified by flash chromatography.

5-(1,2-dithiolan-3-yl)-*N*-(2-oxo-2-(quinolin-8-ylamino)ethyl)pentanamide (**1**).

Purified by chromatography, using CHCl_3_ as the eluent, to obtain pure **1** (37 mg, 0.096 mmol, 69% yield); mp 220–221 °C. The ^1^H NMR (CDCl_3_): δ 1.47–1.55 (m, 2H, CH_2_); 1.68–1.81 (m, 4H, CH_2_); 1.85–1.92 (m, 1H, CH); 2.35 (t, 2H, *J* = 7.6 Hz, CH_2_); 2.40–2.48 (m, 1H, CH); 3.08–3.18 (m, 2H, CH_2_); 3.52–3.58 (m, 1H, CH); 4.29 (d, 2H, *J* = 5.2 Hz, CH_2_); 6.32 (br s, 1H, NH); 7.46–7.49 (m, 1H, Ar); 7.52–7.56 (m, 2H, Ar); 8.17 (dd, 1H, *J* = 1.6, 8.4 Hz, Ar); 8.67–8.71 (m, 1H, Ar); 8.81 (d, 1H, *J* = 3.2 Hz, Ar); and 10.08 (br s, 1H, NH) ppm. The ^13^C NMR (CDCl_3_): δ 173.12, 167.16, 148.53, 138.46, 136.48, 133.91, 128.07, 127.39, 122.19, 121.91, 116.76, 56.45, 44.07, 40.31, 38.57, 36.33, 34.76, 29.01, and 25.42 ppm.

5-(1,2-dithiolan-3-yl)-*N*-(3-oxo-3-(quinolin-8-ylamino)propyl)pentanamide (**2**).

Compound **2** was purified by flash chromatography over silica gel, using CHCl_3_/MeOH (99:1) as the eluent. The solid product was then crystalized from CHCl_3_/hexane (32 mg, 0.08 mmol, 23% yield); mp 100–101 °C. The ^1^H NMR (CDCl_3_): δ 1.38–1.46 (m, 2H, CH_2_); 1.59–1.69 (m, 4H, CH_2_); 1.81–1.88 (m, 1H, CH); 2.18 (t, 2H, *J* = 7.4, CH_2_); 2.34–2.39 (m, 1H, CH); 2.81–2.84 (m, 2H, CH_2_); 3.00–3.14 (m, 2H, CH_2_); 3.45–3.49 (m, 1H, CH); 3.66–3.71 (m, 2H, CH_2_); 6.39 (br s, 1H, NH); 7.45–7.48 (m, 1H, Ar); 7.53–7.54 (m, 2H, Ar); 8.17 (dd, 1H, *J* = 1.6, 8.4 Hz, Ar); 8.71 (dd, 1H, *J* = 2.8, 6 Hz, Ar); 8.80 (dd, 1H, *J* = 1.6, 4.4 Hz, Ar); and 9.83 (br s, 1H, NH) ppm. The ^13^C NMR (CDCl_3_): δ 173.00, 170.54, 148.40, 138.29, 136.46, 134.18, 128.01, 127.31, 121.94, 121.85, 116.53, 56.37, 40.20, 38.47, 37.04, 36.55, 35.41, 34.66, 28.89, and 25.47 ppm.

5-(1,2-dithiolan-3-yl)-*N*-(4-oxo-4-(quinolin-8-ylamino)butyl)pentanamide (**3**).

Compound **3** was purified by flash chromatography over silica gel, using CHCl_3_/MeOH (99:1) as the eluent, and crystallized from CHCl_3_/hexane to afford the final compound **3** (28 mg, 0.07 mmol, 19% yield); mp 184–185 °C. The ^1^H NMR (CDCl_3_): δ 1.33–1.39 (m, 2H, CH_2_); 1.54–1.72 (m, 4H, CH_2_); 1.79–1.86 (m, 1H, CH); 1.99–2.06 (m, 2H, CH_2_); 2.13 (t, 2H, *J* = 7.6 Hz, CH_2_); 2.36–2.41 (m, 1H, CH); 2.65 (t, 2H, *J* = 6.8 Hz, CH_2_); 3.04–3.14 (m, 2H, CH_2_); 3.37–3.42 (m, 2H, CH_2_); 3.46–3.50 (m, 1H, CH); 7.45–7.47 (m, 1H, Ar); 7.50–7.53 (m, 2H, Ar); 8.16 (dd, 1H, *J* = 1.6, 8.0 Hz, Ar); 8.73 (dd, 1H, *J* = 2.2, 6.6 Hz, Ar); 8.80 (dd, 1H, *J* = 1.6, 4.0 Hz, Ar); and 9.85 (br s, 1H, NH) ppm. The ^13^C NMR (CDCl_3_): δ 173.18, 171.68, 148.21, 138.14, 136.60, 134.20, 127.99, 127.37, 121.82, 121.72, 116.69, 56.36, 40.16, 39.28, 38.43, 36.47. 35.57, 34.56, 28.86, 25.38, and 24.86 ppm.

i. General Procedure for the Synthesis of Caffeic Acid Derivatives **4**–**6**

To a solution of commercial caffeic acid (89 mg, 0.49 mmol) in DCM/DMF 1:1, DCC (111 mg, 0.54 mmol), a catalytic amount of DMAP, and amine **12a**–**c** (0.45 mmol) were added. The mixture was stirred for 12 h at rt. Then, the organic solvent was evaporated under vacuum, and the crude product was purified by flash chromatography to afford the final compounds.

(E)-3-(3,4-dihydroxyphenyl)-*N*-(2-oxo-2-(quinolin-8-ylamino)ethyl)acrylamide (**4**)

Compound **4** was purified by gel chromatography using CHCl_3_/MeOH (95:5) as the eluent. Compound **4** (62 mg, 0.17 mmol, 38% yield); mp 229–230 °C. The ^1^H NMR (DMSO-*d*_6_): δ 4.16 (d, 2H, *J* = 6 Hz, CH_2_); 6.52 (d, 1H, *J* = 15.6 Hz, CH=); 6.77 (d, 1H, *J* = 8 Hz, Ar); 6.90 (dd, 1H, *J* = 1.6, 8 Hz, Ar); 7.02 (d, 1H, *J* = 2 Hz, Ar); 7.34 (d, 1H, *J* = 15.6 Hz, CH=); 7.57–7.63 (m, 2H, Ar); 7.68 (dd, 1H, *J* = 1.2, 8.4 Hz, Ar); 8.41 (dd, 1H, *J* = 1.6, 8.4 Hz, Ar); 8.64 (dd, 1H, *J* = 1.2, 8.8 Hz, Ar); 8.72 (br t, 1H, *J* = 6 Hz, NH); 8.85 (dd, 1H, *J* = 1.6, 4 Hz, Ar); and 10.38 (br s, 1H, NH) ppm. The ^13^C NMR (DMSO-*d*_6_): δ 168.32, 166.31, 148.93, 147.54, 145.55, 140.17, 137.89, 136.59, 133.97, 127.78, 126.98, 126.16, 122.19, 121.95, 120.67, 117.52, 116.08, 115.76, 113.87, and 43.87 ppm. HRMS-ESI (*m*/*z*): [M + H]^+^ calcd for [C_20_H_18_N_3_O_4_]^+^ 364.12; found 364.40.

(E)-3-(3,4-dihydroxyphenyl)-*N*-(3-oxo-3-(quinolin-8-ylamino)propyl)acrylamide (**5**)

Compound **5** was purified by flash chromatography by gradient elution with CHCl_3_/MeOH (100:0 to 95:5), affording the final product **5** (17 mg, 0.05 mmol, 14% yield); mp 192–193 °C. The ^1^H NMR (DMSO-*d*_6_): δ 2.80 (t, 2H, *J* = 6.2 Hz, CH_2_); 3.50–3.52 (m, 2H, CH_2_); 6.34 (d, 1H, *J* = 15.6 Hz, CH=); 6.72 (d, 1H, *J* = 8.0 Hz, Ar); 6.81 (d, 1H, *J* = 8.4 Hz, Ar); 6.92 (s, 1H, Ar); 7.23 (d, 1H, *J* = 15.6 Hz, CH=); 7.56–7.68 (m, 3H, Ar); 8.14 (br s, 1H, NH); 8.40 (d, 1H, *J* = 8.0 Hz, Ar); 8.64 (d, 1H, *J* = 7.6 Hz, Ar); 8.90 (d, 1H, *J* = 2.8 Hz, Ar); and 10.14 (br s 1H, NH) ppm. The ^13^C NMR (DMSO-*d*_6_): δ 170.90, 166.38, 149.65, 148.09, 146.33, 139.92, 138.93, 137.40, 135.36, 128.67, 127.77, 127.20, 122.93, 122.71, 121.20, 119.28, 117.61, 116.54, 114.64, 37.70, and 36.16 ppm. HRMS-ESI (*m*/*z*): [M + H]^+^ calcd for [C_21_H_20_N_3_O_4_]^+^ 378,14; found 378.50.

(E)-4-(3-(3,4-dihydroxyphenyl)acrylamido)-*N*-(quinolin-8-yl)butanamide (**6**)

The crude product was purified by flash chromatography over silica gel, using CHCl_3_/MeOH (95:5) as the eluent, affording the final product **6** (61 mg, 0.16 mmol, 33% yield); mp 120–121 °C. The ^1^H NMR (MeOD): δ 2.00–2.07 (m, 2H, CH_2_); 2.67 (t, 2H, *J* = 7.2 Hz, CH_2_); 3.43 (t, 2H, *J* = 6.8 Hz, CH_2_); 6.32 (d, 1H, *J* = 15.6 Hz, CH=); 6.73 (d, 1H, *J* = 8 Hz, Ar); 6.83 (dd, 1H, *J* = 2, 8.4 Hz, Ar); 6.93 (d, 1H, *J* = 2 Hz, Ar); 7.32 (d, 1H, *J* = 15.6 Hz, CH=); 7.50–7.55 (m, 2H, Ar); 7.59 (dd, 1H, *J* = 1.2, 8 Hz, Ar); 8.28 (dd, 1H, *J* = 1.6, 8.4 Hz, Ar); 8.63 (m, 1H, Ar); and 8.86 (dd, 1H, *J* = 1.6, 8 Hz, Ar) ppm. The ^13^C NMR (MeOD): δ 174.03, 169.64, 150.15, 148.95, 146.88, 142.42, 140.25, 137.92, 135.75, 129.85, 128.41, 128.18, 123.73, 123.21, 122.28, 118.72, 118.46, 116.58, 115.17, 40.20, 36.00, and 26.82 ppm.

ii. General Procedure for the Synthesis of Caffeic Acid Derivatives **7** and **8**

To a solution of commercial ferulic acid (60 mg, 0.31 mmol) in DCM/DMF 1:1, DCC (69 mg, 0.34 mmol), a catalytic amount of DMAP, and amine **12a,c** (0.28 mmol) were added. The mixture was stirred for 12 h at rt. Then, the organic solvent was evaporated under vacuum.

(E)-3-(4-hydroxy-3-methoxyphenyl)-*N*-(2-oxo-2-(quinolin-8-ylamino)ethyl)acrylamide (**7**)

The crude product was purified by flash chromatography over silica gel, using CHCl_3_/MeOH (gradient elution from 100:0 to 98:2), affording the final product **7** (16 mg, 0.04 mmol, 15% yield); mp 191–192 °C. The ^1^H NMR (MeOD): δ 3.91 (s, 3H, CH_3_); 4.27 (s, 2H, CH_2_); 6.60 (d, 1H, *J* = 15.6 Hz, CH=); 6.83 (d, 1H, *J* = 8 Hz, Ar); 7.08 (dd, 1H, *J* = 2, 8.4 Hz, Ar); 7.14 (d, 1H, *J* = 1.6Hz, Ar); 7.46–7.59 (m, 4H, 3H Ar + 1H CH=); 8.23 (dd, 1H, *J* = 1.2, 8.4 Hz, Ar); 8.63–8.66 (m, 1H, Ar); and 8.76 (dd, 1H, *J* = 1.6, 4.4 Hz, Ar) ppm. The ^13^C NMR (MeOD): δ 169.51, 169.43, 149.58, 149.56, 148.85, 143.02, 139.56, 137.25, 134.55. 129.09, 127.70, 123.29, 123.17, 122.67, 117.66, 117.53, 116.20, 111.31, 56.24, and 44.98 ppm.

(E)-4-(3-(4-hydroxy-3-methoxyphenyl)acrylamido)-*N*-(quinolin-8-yl)butanamide (**8**)

The crude product was purified flash chromatography over silica gel, using CHCl_3_/MeOH (95:5) as the eluent, affording the final product **8** (36 mg, 0.09 mmol, 24% yield); mp 177–177 °C. The ^1^H NMR (CDCl_3_): δ 2.07–2.14 (m, 2H, CH_2_); 2.70 (t, 2H, *J* = 6.8 Hz, CH_2_); 3.53–3.58 (m, 2H, CH_2_); 3.90 (s, 3H, CH_3_); 5.81 (s, 1H, Ar); 6.20 (d, 1H, *J* = 15.6 Hz, CH=); 6.87 (d, 1H, *J* = 8 Hz, Ar); 6.91 (d, 1H, *J* = 2, Ar); 6.96 (dd, 1H, *J* = 2, 8.4 Hz, Ar); 7.43–7.54 (m, 4H, 3H Ar + 1H CH=); 8.13 (dd, 1H, *J* = 1.6, 8.4 Hz, Ar); 8.75 (dd, 1H, *J* = 1.6, 7.2 Hz, Ar); 8.80 (dd, 1H, *J* = 1.6, 8 Hz, Ar); and 9.87 (br s, 1H, NH) ppm. The ^13^C NMR (CDCl_3_): δ 171.73, 166.52, 148.30, 147.30, 146.67, 140.77, 138.37, 136.41, 134.37, 128.01, 127.45, 127.30, 122.22, 121.77, 121.73, 118.41, 116.55, 114.65, 109.48, 55.95, 39.47, 35.65, and 25.06 ppm.

iii. Synthesis of Intermediates **10a**–**c**, **11a**–**c**, and Free Amines **12a**–**c**

(Tert-butoxycarbonyl)glycine (**10a**)

To a solution of commercial glycine **9a** (1.00 g, 13.32 mmol) in NaOH 1M/iPrOH 4:3 at 0 °C, Boc_2_O (2.91 g, 13.32 mmol) was added. The reaction, monitored by TLC, was stirred at rt for 2 h, washed with Et_2_O, acidified to pH 3.0 with 1N HCl, and, finally, extracted with CHCl_3_. The organic layer was dried over anhydrous Na_2_SO_4_ and evaporated under reduced pressure. The crude product (1.24 g, 7.06 mmol, 53% yield) was used for the next step, without further purification. The ^1^H NMR (CDCl_3_): δ 1.45 (s, 9H, CH_3_); 3.94–3.96 (m, 2H, CH_2_) ppm.

3-((tert-butoxycarbonyl)amino)propanoic acid (**10b**)

To a solution of commercial L-alanine _9b_ (1.00 g, 11.22 mmol) in NaOH 1M/iPrOH 4:3 at 0 °C, Boc_2_O (2.45 g, 11.22 mmol) was added. The procedure followed was the same as that described for derivative **10a**. The crude product (1.23 g, 6.51 mmol, 58% yield) was used for the next step, without further purification. The ^1^H NMR (CDCl_3_): δ 1.43 (s, 9H, CH_3_); 2.58 (m, 2H, CH_2_); and 3.39–3.41 (m, 2H, CH_2_) ppm.

4-((tert-butoxycarbonyl)amino)butanoic acid (**10c**)

To a solution of commercial γ-aminobutyric acid (1.00 g, 11.22 mmol) in NaOH 1M/iPrOH 4:3 at 0 °C, Boc_2_O (2.45 g, 11.22 mmol) was added. The procedure followed was the same as that described for derivative **10a**. The crude product (1.32 g, 6.51 mmol, 58% yield) was used for the next step, without further purification. The ^1^H NMR (CDCl_3_): δ 1.43 (s, 9H, CH_3_); 1.79–1.83 (m, 2H, CH_2_); 2.39 (t, 2H, *J* = 7.2 Hz, CH_2_); and 3.17–3.19 (m, 2H, CH_2_) ppm.

Tert-butyl (2-oxo-2-(quinolin-8-ylamino)ethyl)carbamate (**11a**)

To a solution of compound **10a** (400 mg, 2.29 mmol), in dry DMF, under N_2_ atmosphere, and cooled to 0 °C, TBTU (735 mg, 2.29 mmol) and DIPEA (0.8 mL, 4.58 mmol) were added. After 30 min at 0 °C, commercial 8-aminoquinoline (330 mg, 2.29 mmol) was added, and the temperature was kept at 0 °C for an additional 30 min. Later, the mixture was slowly warmed to rt and left under stirring at rt for 72 h. Then, the organic solvent was reduced under vacuum, and the crude residue was diluted with DCM and washed with H_2_O and a solution of HCl 10%. The organic layer was dried over Na_2_SO_4_, and the solvent was evaporated, affording the intermediate **11a** that was used for the next step, without further purification (621 mg, 2.06 mmol, 90% yield). The ^1^H NMR (DMSO-*d*_6_): δ 1.45 (s, 9H, CH_3_); 3.83 (d, 2H, *J* = 6 Hz, CH_2_); 7.53–7.77 (m, 4H, 3H Ar + 1H NH); 8.42 (dd, 1H, *J* = 1.6, 8.4 Hz, Ar); 8.65 (d, 1H, *J* = 6.4 Hz, Ar); 8.89 (dd, 1H, *J* = 1.6, 4.4 Hz, Ar); and 10.43 (br s, 1H, NH) ppm.

Tert-butyl (3-oxo-3-(quinolin-8-ylamino)propyl)carbamate (**11b**)

To a solution of compound **10b** (400 mg, 2.11 mmol), in dry DMF, under N_2_ atmosphere, and cooled to 0 °C, TBTU (677 mg, 2.11 mmol) and DIPEA (0.7 mL, 4.22 mmol) were added. After 30 min at 0 °C, commercial 8-aminoquinoline (306 mg, 2.11 mmol) was added, and the temperature was kept at 0 °C for an additional 30 min. Later, the mixture was slowly warmed to rt and left under stirring at rt for 72 h. Then, the organic solvent was reduced under vacuum, and the crude residue was diluted with DCM and washed with H_2_O and a solution of HCl 10%. The organic layer was dried over Na_2_SO_4_, and the solvent was evaporated, affording the intermediate **11b** that was used for the next step, without further purification (313 mg, 0.99 mmol, 47% yield). The ^1^H NMR (CHCl_3_): δ 1.47 (s, 9H, CH_3_); 2.88 (t, 2H, *J* = 6.0 Hz, CH_2_); 3.55–3.57 (m, 2H, CH_2_); 7.63–7.65 (m, 3H, Ar); 8.38 (d, 1H, *J* = 8.0 Hz, Ar); 8,79 (d, 1H, *J* = 8.0 Hz, Ar); 8.86 (d, 1H, *J* = 3.6 Hz, Ar); and 10,13 (br s, 1H, NH) ppm.

Tert-butyl (4-oxo-4-(quinolin-8-ylamino)butyl)carbamate (**11c**)

To a solution of compound **10c** (400 mg, 1.97 mmol), in dry DMF, under N_2_ atmosphere, and cooled to 0 °C, TBTU (632 mg, 1.97 mmol) and DIPEA (0.7 mL, 3.94 mmol) were added. After 30 min at 0 °C, commercial 8-aminoquinoline (285 mg, 1.97 mmol) was added, and the temperature was kept at 0 °C for an additional 30 min. Later, the mixture was slowly warmed to rt and left under stirring at rt for 72 h. Then, the organic solvent was reduced under vacuum, and the crude residue was treated with H_2_O. The resulting precipitate was collected by filtration and dried, affording the intermediate **11c** that was used for the next step, without further purification (552 mg, 1.67 mmol, 85% yield). The ^1^H NMR (DMSO-*d*_6_): δ 1.37 (s, 9H, CH_3_); 1.73–1.78 (m, 2H, CH_2_); 2.57 (t, 2H, *J* = 7.2 Hz, CH_2_); 2.97–3.02 (m, 2H, CH_2_); 6.89 (br t, 1H, *J* = 5.6 Hz, NH); 7.57 (t, 1H, *J* = 7.6 Hz, Ar); 7.62–7.67 (m, 2H, Ar); 8.41 (dd, 1H, *J* = 1.6, 8.4 Hz, Ar); 8.63 (dd, 1H, *J* = 1.2, 8.4 Hz, Ar); 8.93 (dd, 1H, *J* = 1.6, 8.4 Hz, Ar); and 10.07 (br s, 1H, NH) ppm.

2-amino-*N*-(quinolin-8-yl)acetamide (**12a**)

To a stirred solution of **11a** (138 mg, 0.46 mmol) in DCM, cooled at −10 °C/−20 °C, TFA (0.916 mL) was added. The reaction was monitored by TLC and reached completion in 3 h at the same temperature. Then, the reaction mixture was quenched with H_2_O, and the organic solvent was evaporated under vacuum. The aqueous layer was alkalinized with NaOH 1N and extracted with DCM. The organic layer was dried over Na_2_SO_4_, and the solvent was evaporated, affording the free amine **12a** (54 mg, 0.27 mmol, 58% yield). The ^1^H NMR (DMSO-*d*_6_): δ 3.39 (s, 2H, CH_2_); 7.55–7.66 (m, 3H, Ar); 8.40 (dd, 1H, *J* = 1.6, 8.4 Hz, Ar); 8.74 (dd, 1H, *J* = 1.2, 7.6 Hz, Ar); 8.92 (dd, 1H, *J* = 1.6, 4 Hz, Ar); and 11.62 (br s, 1H, NH) ppm.

3-amino-*N*-(quinolin-8-yl)propenamide (**12b**)

To a stirred solution of **11b** (330 mg, 1.04 mmol) in DCM, cooled at −10 °C/−20 °C, TFA (2.08 mL) was added. The reaction was monitored by TLC and reached completion in 3 h at the same temperature. Then, the reaction mixture was quenched with H_2_O, and the organic solvent was evaporated under vacuum. The aqueous layer was alkalinized with NaOH 1N and extracted with DCM. The organic layer was dried over Na_2_SO_4_, and the solvent was evaporated, affording the free amine **12b** (139 mg, 0.64 mmol, 62% yield). The ^1^H NMR (CDCl_3_): δ 2.72 (t, 2H, *J* = 6 Hz, CH_2_); 3.19 (t, 2H, *J* = 6 Hz, CH_2_); 7.43–7.46 (m, 1H, Ar); 7.48–7.55 (m, 2H, Ar); 8.15 (dd, 1H, *J* = 1.6, 8.4 Hz, Ar); 8.77 (dd, 1H, *J* = 1.6, 7.0 Hz, Ar); 8.80 (dd, 1H, *J* = 1.6, 4.4 Hz, Ar); and 10.21 (br s, 1H, NH) ppm.

4-amino-*N*-(quinolin-8-yl)butanamide (**12c**)

To a stirred solution of **11c** (347 mg, 1.05 mmol) in DCM, cooled at −10 °C/−20 °C, TFA (2.11 mL) was added. The reaction was monitored by TLC and reached completion in 3 h at the same temperature. Then, the reaction mixture was quenched with H_2_O, and the organic solvent was evaporated under vacuum. The aqueous layer was alkalinized with NaOH 1N and extracted with DCM. The organic layer was dried over Na_2_SO_4_, and the solvent was evaporated, affording the free amine **12c** (164 mg, 0.71 mmol, 68% yield). The ^1^H NMR (DMSO-*d*_6_): δ 1.70–1.77 (m, 2H, CH_2_); 2.59–2.65 (m, 4H, CH_2_); 7.57 (t, 1H, *J* = 8 Hz, Ar); 7.62–7.66 (m, 2H, Ar); 8.40 (dd, 1H, *J* = 1.6, 8 Hz, Ar); 8.63 (dd, 1H, *J* = 1.2, 7.6 Hz, Ar); 8.92 (dd, 1H, *J* = 1.6, 8.4 Hz, Ar); and 10.08 (br s, 1H, NH) ppm.

### 4.2. In Vitro Biological Assay

#### 4.2.1. Metal-Chelating Study

The copper-chelating activity was performed as previously reported and monitored spectrophotometrically using a UV-Vis PerkinElmer EnSpire 2300 spectrophotometer [27]. CuCl_2_ was dissolved in absolute ethanol to make a concentration of 400 μM. Compounds **1**–**8** were dissolved in 100% DMSO and diluted in absolute ethanol to reach the final concentrations of 20 μM. Then, 100 µL of each compound solution was diluted with absolute ethanol to obtain 100, 50, 25, 12.5, 6.25, 3.125, 1.56, and 0 µM concentrations. Then, 100 µL of the CuCl_2_ solution was added to make a final concentration of compounds of 10 µM, which were incubated at room temperature for 30 min. Then, the absorption spectrum was recorded with a UV–Vis spectrophotometer.

#### 4.2.2. DPPH Assay

DPPH was dissolved in methanol to make a concentration of 1 mM, and compounds **1**–**8** were dissolved in methanol and diluted in a 96-well plate at several concentrations (200, 100, and 50 μM) [27]. Then, in each well, the DPPH solution was added (final concentration of 500 nM) and incubated at room temperature in the dark for 30 min. The absorbance was read at 531 nm with a PerkinElmer EnSpire 2300 multiplate reader. The capacity of scavenging free radicals was calculated as followed: capacity = (A_DDPH_ − A_compound_/A_DPPH_) × 100, where A_DPPH_ and A_compound_ are, respectively, the absorbances of the control and the tested compounds. Instead, to detect the IC_50_ of the selected compounds, 300 µM methanolic solution of compounds was properly diluted to 150, 75, 37.5, 18.8, 9.4, 4.7, and 0 µM (duplicated), following the procedure previously described.

### 4.3. In Vitro Cell Line Screening

#### 4.3.1. Cell Culture

The 661W photoreceptor cells were supplied by Dr. Muayyad Al-Ubaidi (University of Oklahoma Health Sciences Center). These cells were cultured in Dulbecco’s Modified Eagle’s medium, high glucose, (DMEM) with 10% (*v*/*v*) fetal bovine serum and 1% penicillin–streptomycin solution and maintained at 37 °C in a humidified incubator with 5% CO_2_. The material used for the cell cultures was purchased from Sigma-Aldrich (Merck, Darmstadt, Germany).

#### 4.3.2. Drug Stock Preparation

The hydrogen peroxide solution (H_2_O_2_) was purchased from Sigma-Aldrich and diluted to a working concentration in DMEM. The compounds were synthesized as described above, and stock solutions (10 mM) were prepared in DMSO and then diluted to working concentrations in DMEM.

#### 4.3.3. Cell Viability

Cell vitality was tested by using CellTiter 96 AQueous One Solution Reagent (Promega, WI USA). The cells were seeded in a 96-well plate at a density of 5 × 10^3^ cells per well and incubated overnight at 37 °C in 5% CO_2_. The cells were then pre-treated with the compounds (1–100 µM) for 24 h. The next day, H_2_O_2_ was added at the concentration of 500 µM and, after 3 h, the cells were treated with the One Solution Reagent and further incubated for 2 h at 37 °C, 5% CO_2_. The absorbance was measured at 490 nm with a 96-well plate reader. The quantity of the formazan product, as measured by the amount of 490 nm absorbance, was directly proportional to the number of living cells in the culture. The percentage of cell viability was normalized to the control group (no compounds or H_2_O_2_ exposure).

#### 4.3.4. Intracellular Reactive Oxygen Species’ (ROS) Determination

The cells undergoing oxidative stress, defined by the presence of ROS, namely, superoxide, were determined by a Muse^®^ Oxidative Stress Kit. Briefly, after culturing, in a 24-well plate at a density of 5 × 10^4^ cells per well, the cells were pre-treated with compounds **4** and **5** at the chosen concentration of 100 µM. The next day, H_2_O_2_ was added at the concentration of 500 µM, and, after 3 h, the cells were re-suspended at a concentration of 1 × 10^6^ cells per mL in 1X assay buffer (Muse^®^ Oxidative Stress Kit). After that, the samples were incubated for 30 min at 37 °C, and then the ROS-positive cells were examined using the Muse^®^ Cell Analyzer.

#### 4.3.5. Immunofluorescence

The cells were seeded onto an eight-well chamber slide at a density of 10^4^ cells/well. The cells were pre-treated, with compounds **4** and **5**, as indicated, fixed in 2% paraformaldehyde for 15 min, permeabilized with 2.5% bovine serum albumin (BSA) and 0.3% Triton X-100 for 10 min, blocked in 2.5% BSA for 1 h, then incubated overnight at 4 °C with primary antibodies against cone arrestin (Millipore, ab15282, 1:1000) and acrolein (Abcam, ab48501, 1:1000). The next day, the cells were incubated with Anti-Rabbit Alexa Flour-488 and Anti-Mouse Alexa Flour-568 for 2 h at room temperature. Finally, the cells were counterstained with DAPI and washed three times with PBS, and images were acquired by using a Nikon Ni-E confocal microscope using a 20X objective. For each treatment group, a total of 50 cells, distributed in three different fields, were measured using a 20X objective. The acrolein signal was calculated as “correct total cell fluorescence” (CTFC), based on the signal intensity and area of each individual cell, to normalize the signal distribution within the cells with different sizes. Each cell was contoured to the cell membrane using bright-field images. Then, each cell’s area and integrated density, as well as five measurements of the surrounding background, were calculated using ImageJ software. CTCF was calculated as follows: CTCF = integrated density—(selected cell area × average background density). Values were subsequently normalized with the H_2_O_2_ 500 µM group.

#### 4.3.6. Statistical Analysis in Vitro Assay

The Origin Lab 8.0 program (MicroCal, Northampton, MA, USA) was used for data analysis and graphical presentation. All data are presented as the means ± SE. Statistical analyses were performed by using a one-way ANOVA test followed by Levene’s post-test, as indicated in each graphic. The *p*-value < 0.05 was considered to be statistically significant.

## 5. Conclusions

Neurodegenerative and orphan diseases include a wide range of pathologies, which share a very complex etiology, resulting in a lack of effective treatments to solve or at least relieve patients’ symptomatology. In the last decades, accumulating evidence indicated a key role for ROS-dependent cellular damage in many cellular dysfunctions, also linked to abnormal metal accumulation. Our findings indicate that 8-aminoquinoline derivatives 4 and 5 significantly reduce oxidative stress in a photoreceptor-like cell line, supporting that the synthesis of molecules with dual antioxidant and chelating properties may be promising MTDL candidates to fight OS. This activity is particularly important in relation to neurodegenerative diseases, such as RP, where the progression of the pathology is driven by multifactorial events including the increase of OS leading to secondary degeneration and the death of cones, the photoreceptors essential for daytime vision in humans, which is a priority to protect in order to preserve a residual visual function in patients. Our work supports the rationale that opportunely designed pleiotropic agents, synthesized following the MTDL approach, could offer new opportunities for the identification of innovative and effective therapies against OS.

## Figures and Tables

**Table 1:** Scavenging activity of **1**–**8** and LA, FA, and CA. Antioxidant activity is expressed as % of antiradical potential towards a 500 µM of DPPH ± SD. The results reported are the mean ± SD of two independent experiments.

	Percentage Radical Scavenging (%)										
Concetration	LA	1	2	3	CA	4	5	6	FA	7	8
200 μM	3.2 ± 5.2	−5.8 ± 1.3	26.9 ± 2.6	4.1 ± 0.2	63.9 ± 5.0	85.9 ± 1.6	90.4 ± 0.7	79.8 ± 0.4	31.0 ± 3.4	26.6 ± 5.3	36.9 ± 0.4
100 μM	−3.1 ± 10	−11.6 ± 1.1	19.5 ± 2.7	−2.8 ± 0.1	41.5 ± 3.9	76.5 ± 6.6	78.6 ± 8.4	46.5 ± 2.1	20.3 ± 2.1	16.0 ± 0.5	26.5 ± 2.5
50 μM	−2.1 ± 1.3	−11.4 ± 1.0	26.7 ± 6.1	−5.1 ± 5.9	23.2 ± 3.2	42.3 ± 12.2	44.0 ± 1.8	33.9 ± 0.8	13.2 ± 0.5	9.9 ± 1.3	23.5 ± 1.4

## Data Availability

Data is contained within the article and Supplementary Materials.

## Supplementary Materials

The following supporting information can be downloaded at https://www.mdpi.com/article/10.3390/ph15060688/s1. Figure S1. Copper chelating study; DPPH assay. Cell viability was analyzed by CellTiter 96 AQueous One Solution Reagent. Cells were pre-treated for 24 h with compounds at various concentrations (1–100 µM) and then exposed for 3 h with H_2_O_2_ 500 µM. The dashed line indicates the reference value of Ctrl (control group, no compounds or H_2_O_2_ exposure). Values in the graph indicate % viability as the mean ± SE obtained from *n* = 3 of independent experiments; ^1^H-NMR and ^13^C-NMR spectra of final products **1–8**; HPLC spectra of final compounds **1–8**.

## Abbreviations

DCC: *N,N*′-Dicyclohexylcarbodiimide; DCM: dichloromethane; DIPEA: *N,N*-Diisopropylethylamine; DMAP: *N,N*-Dimethylpyridin-4-amine; DMF: *N,N*-dimethylformamide; TBTU: 2-(1*H*-Benzotriazole-1-yl)-1,1,3,3-tetramethylaminium tetrafluoroborate; TFA: trifluoroacetic acid.
