# Supplementary material for: Design, Synthesis, and In Vitro Evaluation of Novel 8-Amino-Quinoline Combined with Natural Antioxidant Acids
Source: Pharmaceuticals (Basel). 2022 May 31;15(6):688. doi: 10.3390/ph15060688 (PMC9229476; doi:10.3390/ph15060688)
Supplement: Supplementary file 1 [file pharmaceuticals-15-00688-s001.zip › pharmaceuticals-1680685-supplementary.pdf]

pharmaceuticals

MDPI

Article

Design, synthesis and in vitro evaluation of novel 8-amino-quinoline combined with natural antioxidant acids

Andrea Bacci[1], Francesca Corsi[1], Massimiliano Runfola[1], Simona Sestito[2], Ilaria Piano[1], Clementina Manera[1], Giuseppe Saccomanni[1], Claudia Gargini[1], Simona Rapposelli [*1,3]

1. Department of Pharmacy, University of Pisa, Via Bonanno 6, 56126 Pisa, Italy
2. Department of Chemistry and Pharmacy, University of Sassari, Via Vienna 2, 07100 Sassari
3. CISUP, Center for Instrument Sharing, University of Pisa, 56126 Pisa, Italy

* Corresponding Authors: S.R. simona.rapposelli@unipi.it

Contents

^{1}H-NMR and ^{13}C NMR spectra of 1-8 .... S1-S9

HPLC Analysis of 1-8.......... S10

Copper chelating study S18

DPPH assay S18

Cell viability S19

1. ^{1}H-NMR AND ^{13}C-NMR SPECTRA OF FINAL PRODUCTS 1-8

5-(1,2-dithiolan-3-yl)-N-(2-oxo-2-(quinolin-8-ylamino)ethyl)pentanamide (1)

^{1}H-NMR

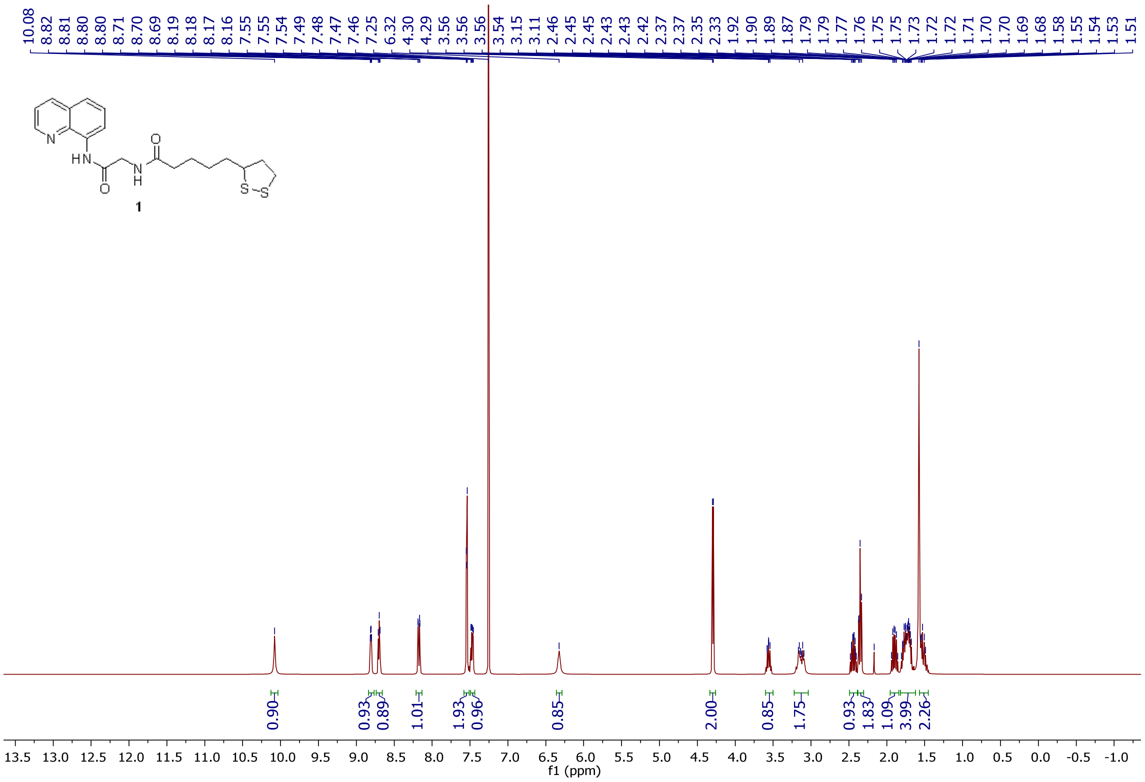

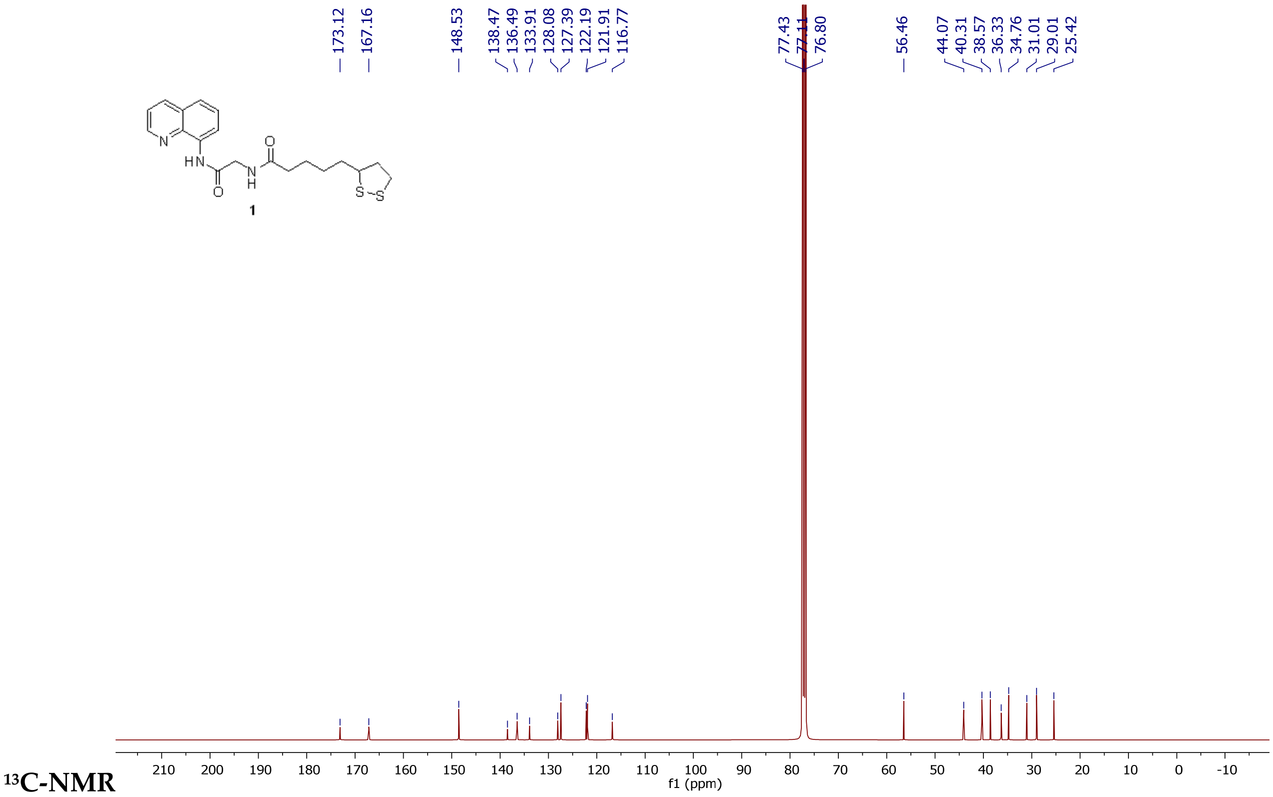

^{13}C-NMR

5-(1,2-dithiolan-3-yl)-N-(3-oxo-3-(quinolin-8-ylamino)propyl)pentanamide (2)

^{1}H-NMR

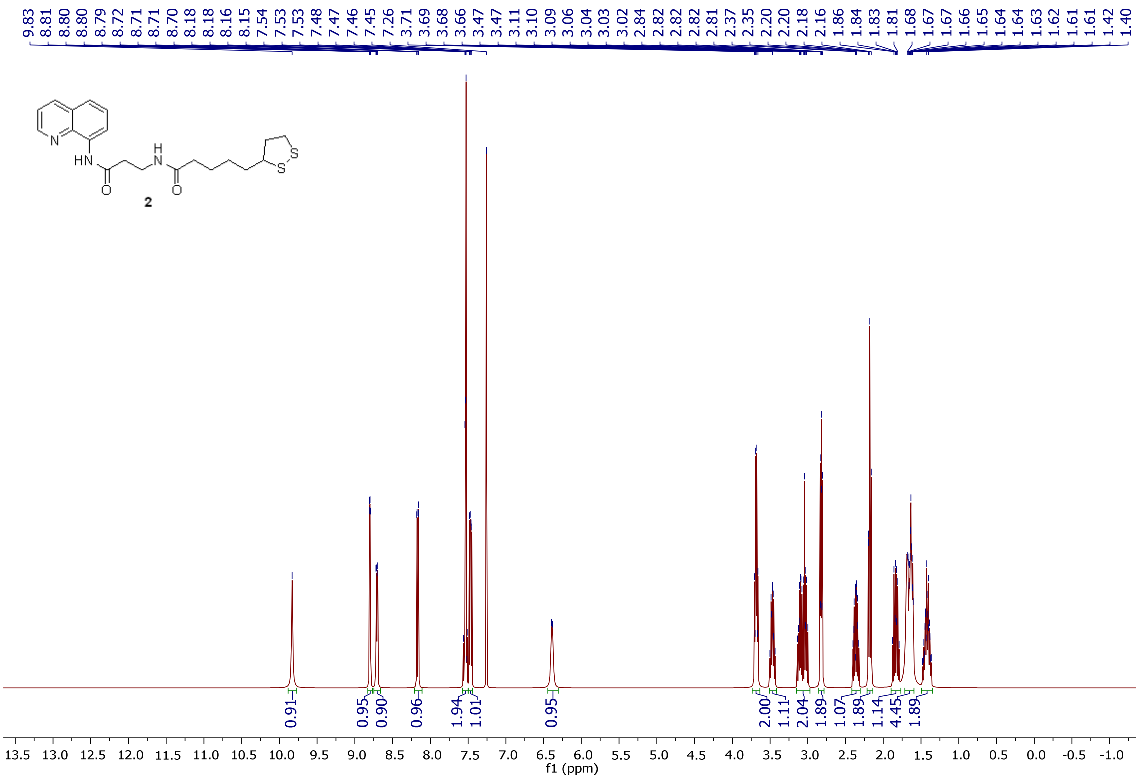

^{13}C-NMR

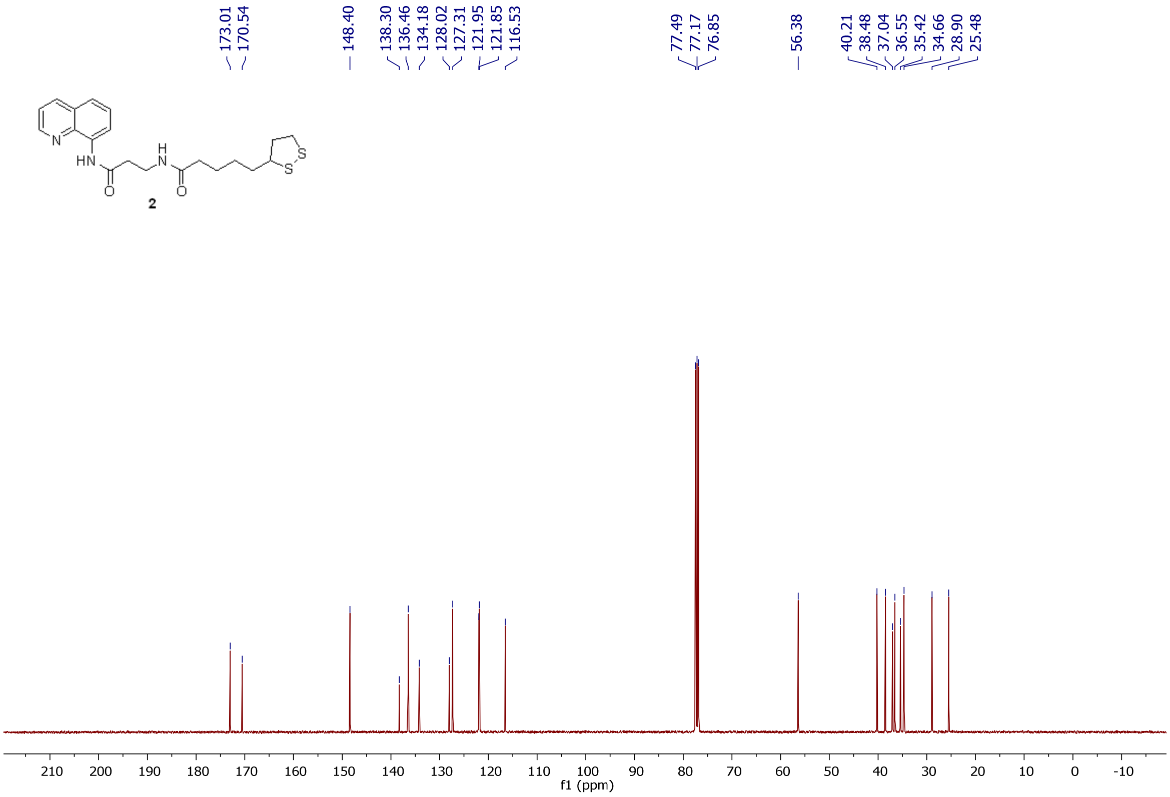

5-(1,2-dithiolan-3-yl)-N-(4-oxo-4-(quinolin-8-ylamino)butyl)pentanamide (3)

^{1}H-NMR

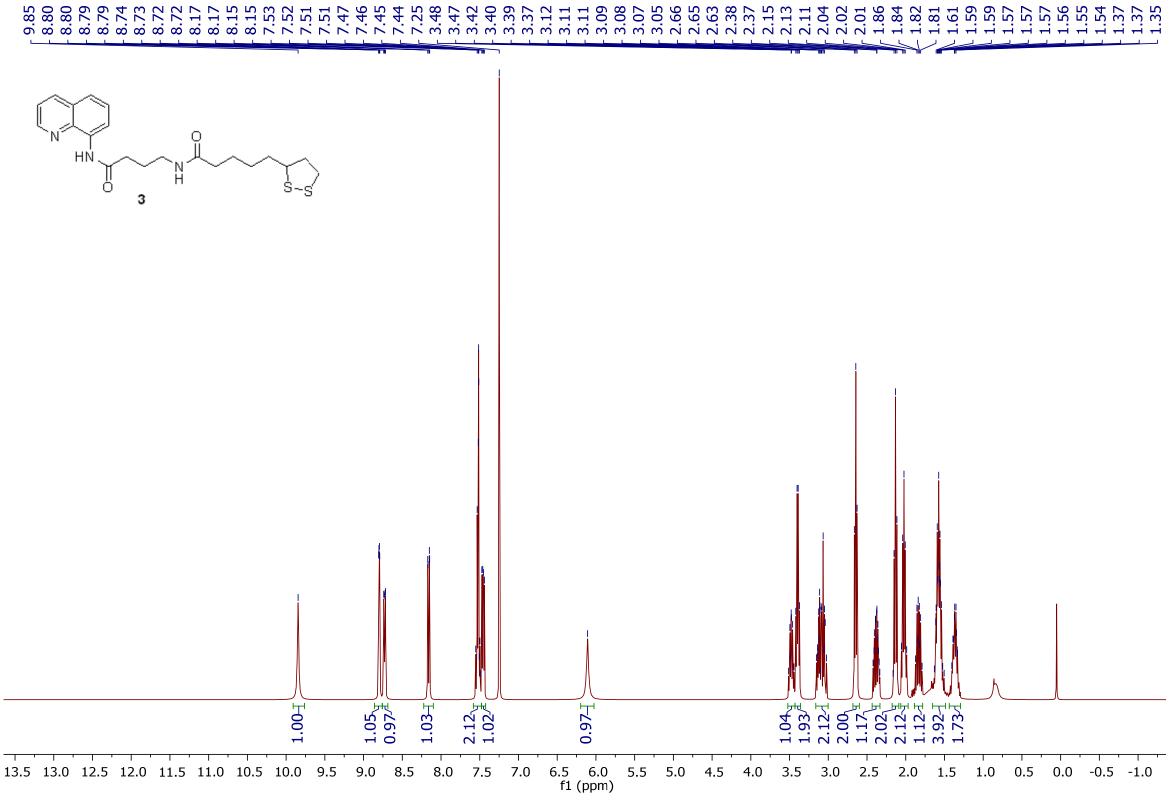

^{13}C-NMR

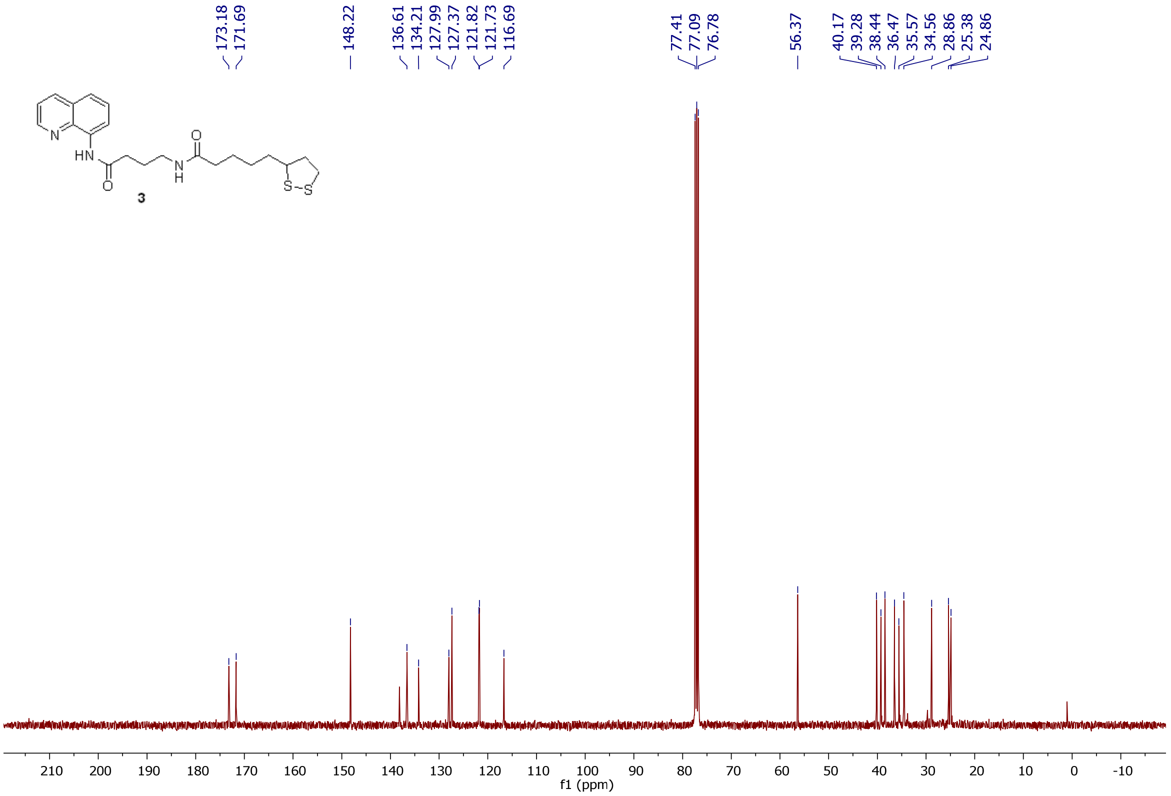

(E)-3-(3,4-dihydroxyphenyl)-N-(2-oxo-2-(quinolin-8-ylamino)ethyl)acrylamide (4)

^{1}H-NMR

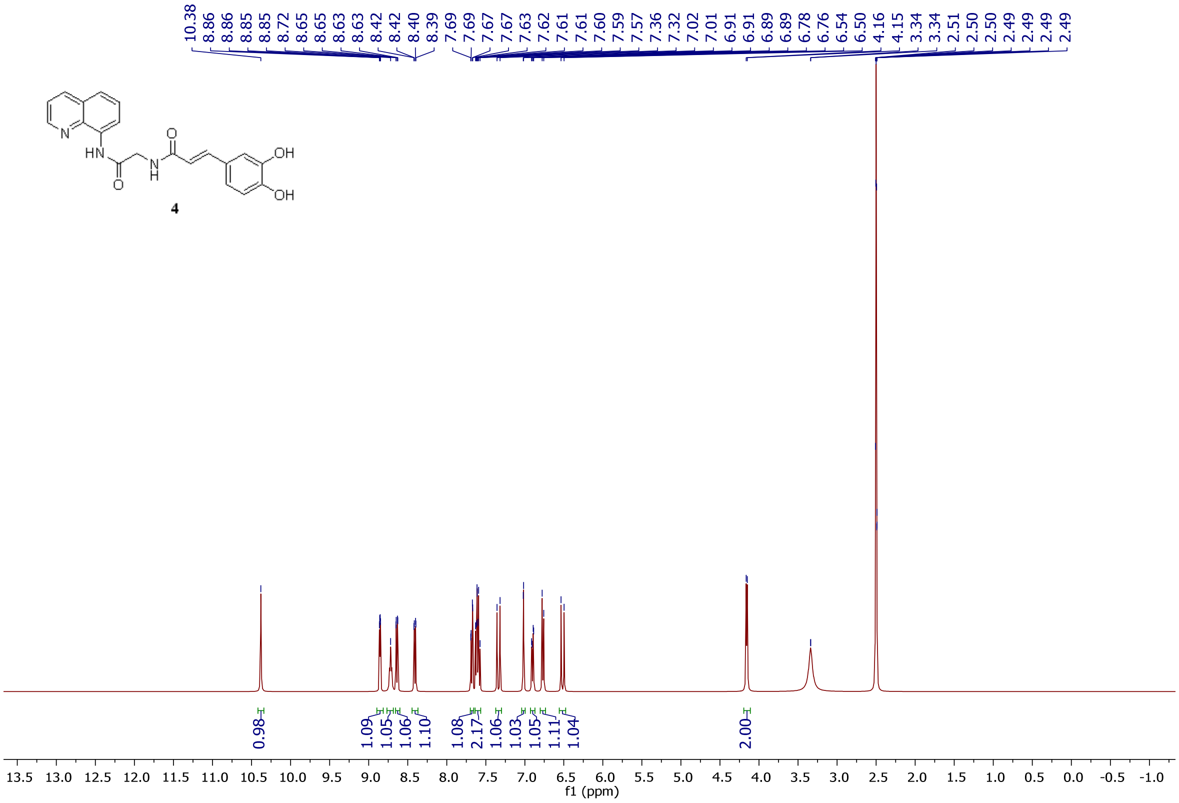

^{13}C-NMR

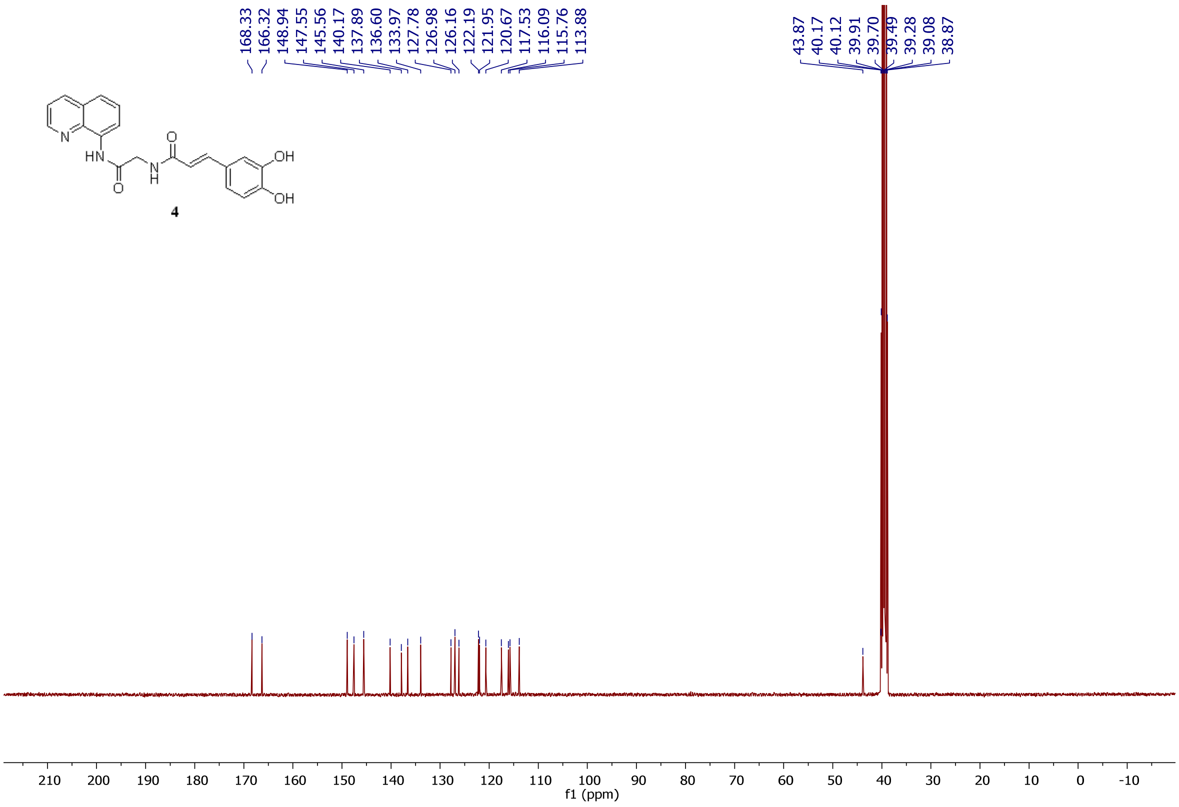

(E)-3-(3,4-dihydroxyphenyl)-N-(3-oxo-3-(quinolin-8-ylamino)propyl)acrylamide (5)

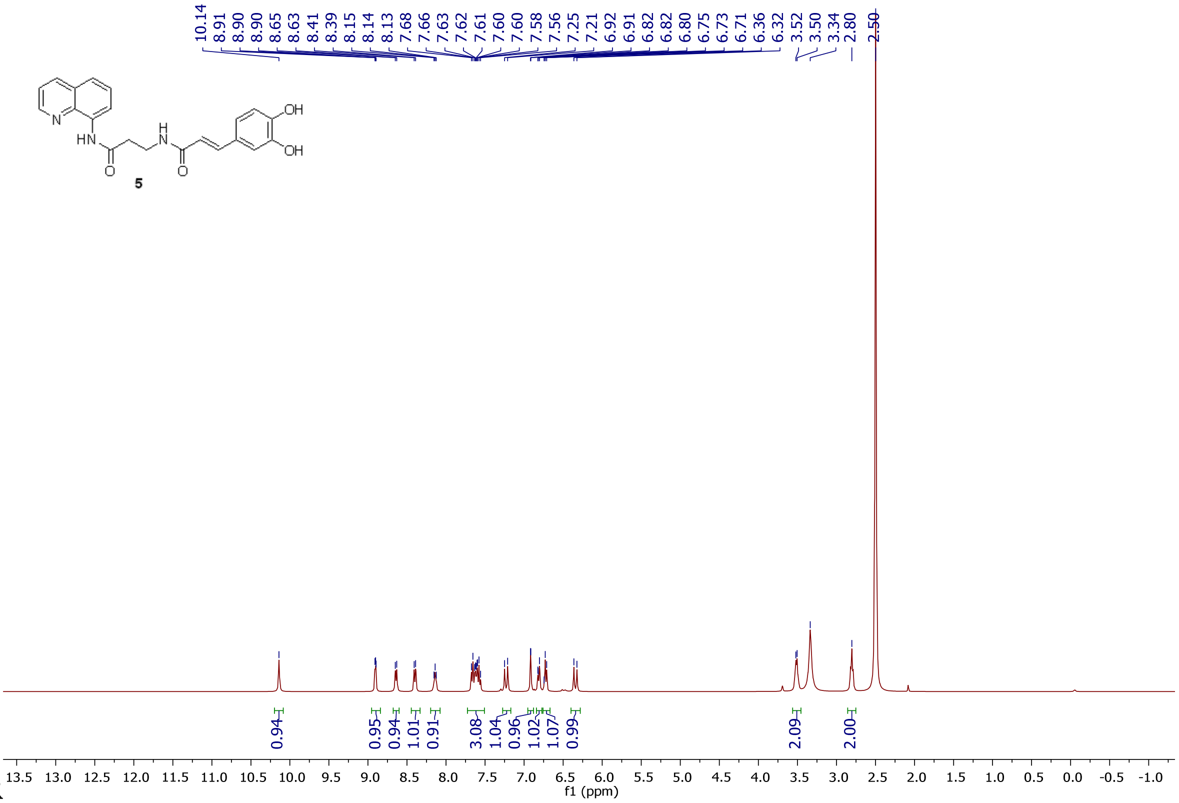

^{1}H-NMR

^{13}C-NMR

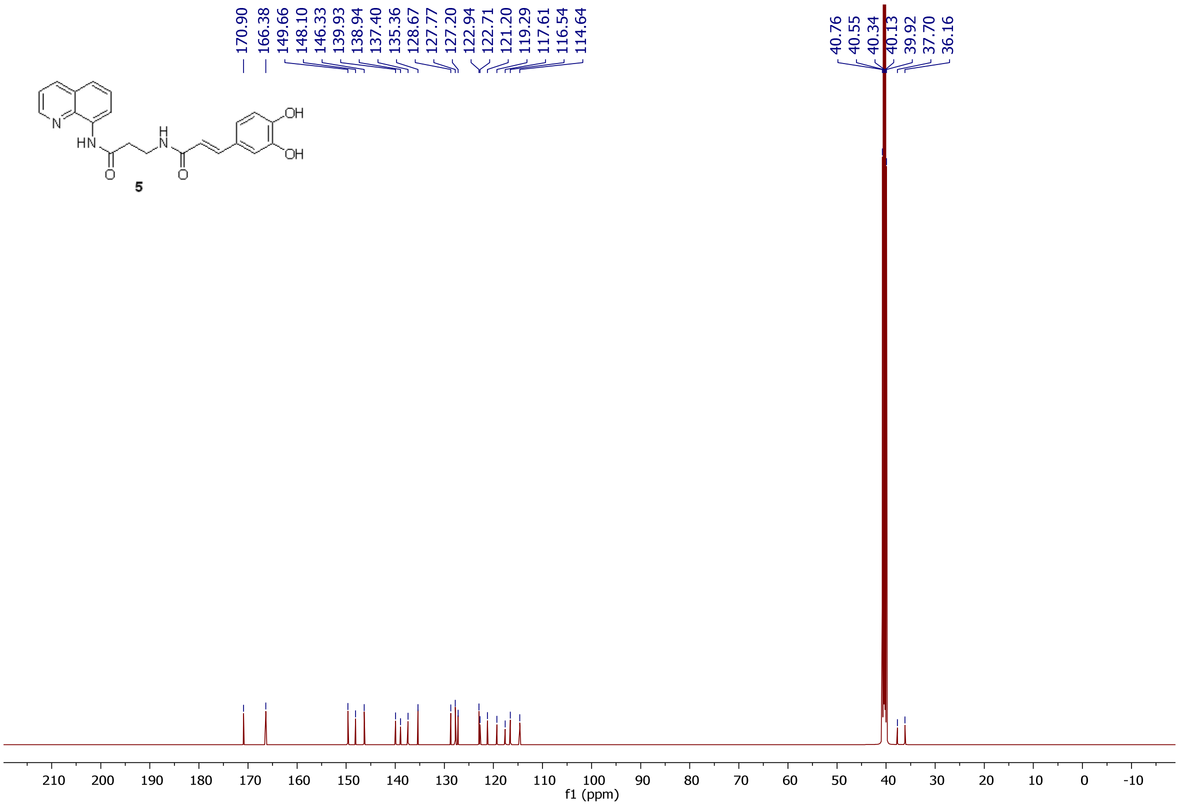

(E)-4-(3-(3,4-dihydroxyphenyl)acrylamido)-N-(quinolin-8-yl)butanamide (6)

^{1}H-NMR

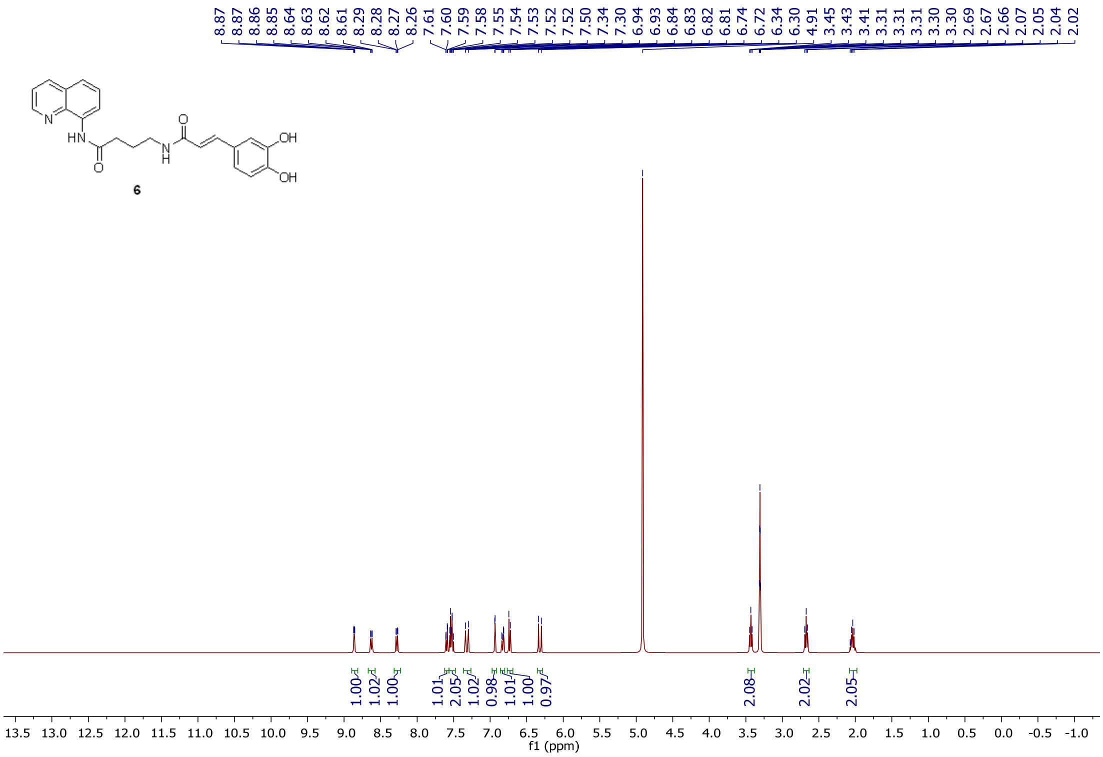

^{13}C-NMR

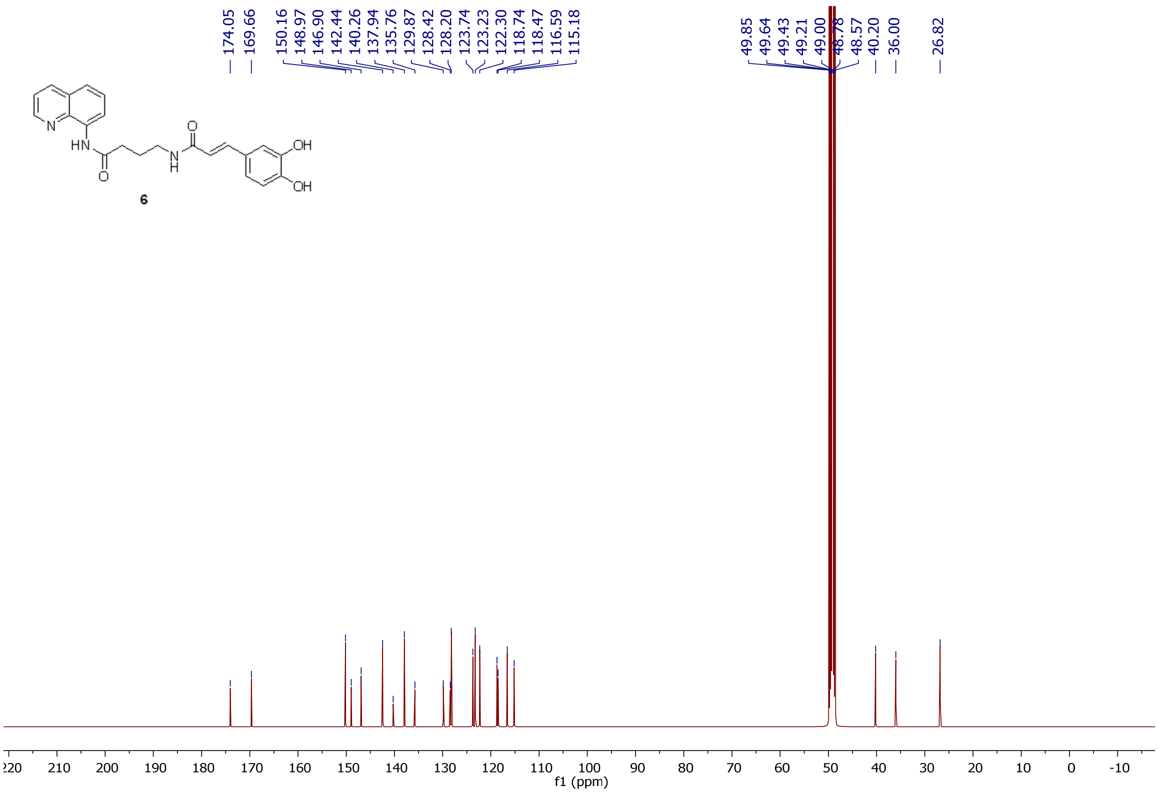

(*E*)-3-(4-hydroxy-3-methoxyphenyl)-N-(2-oxo-2-(quinolin-8-ylamino)ethyl)acrylamide **(7)**

^{1}H-NMR

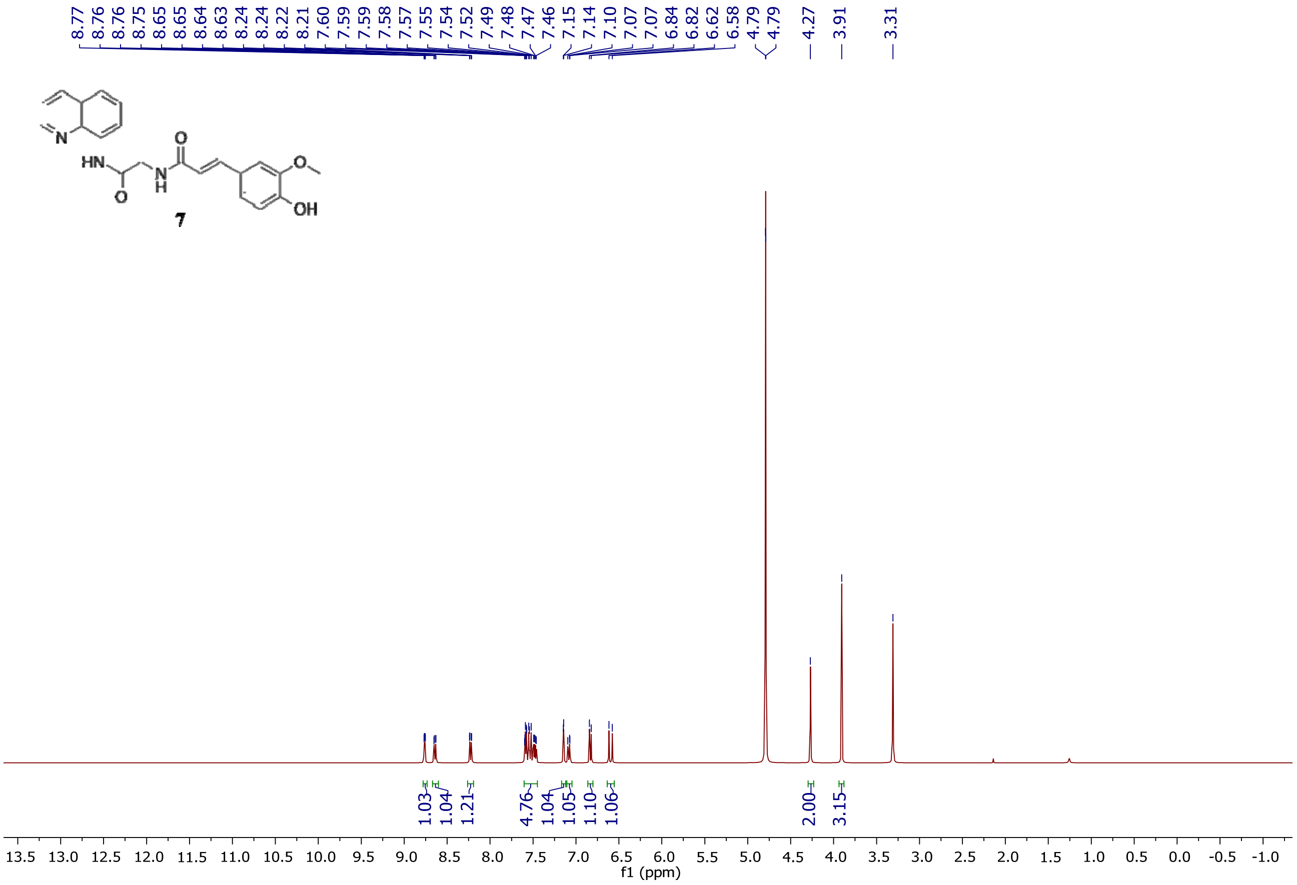

8.77 8.76 8.76 8.75 8.65 8.65 8.64 8.63 8.24 8.24 8.22 8.21 7.60 7.59 7.59 7.58 7.57 7.55 7.54 7.52 7.49 7.48 7.47 7.46 7.15 7.14 7.10 7.07 7.07 6.84 6.82 6.62 6.58 4.79 4.79
4.27
3.91
3.31
N
HN
O
N
H
O
O
OH
7
1.03 1.04 1.21 4.76 1.04 1.05 1.10 1.06
2.00 3.15
13.5 13.0 12.5 12.0 11.5 11.0 10.5 10.0 9.5 9.0 8.5 8.0 7.5 7.0 6.5 6.0 5.5 5.0 4.5 4.0 3.5 3.0 2.5 2.0 1.5 1.0 0.5 0.0 -0.5 -1.0
f1 (ppm)

^{13}C-NMR

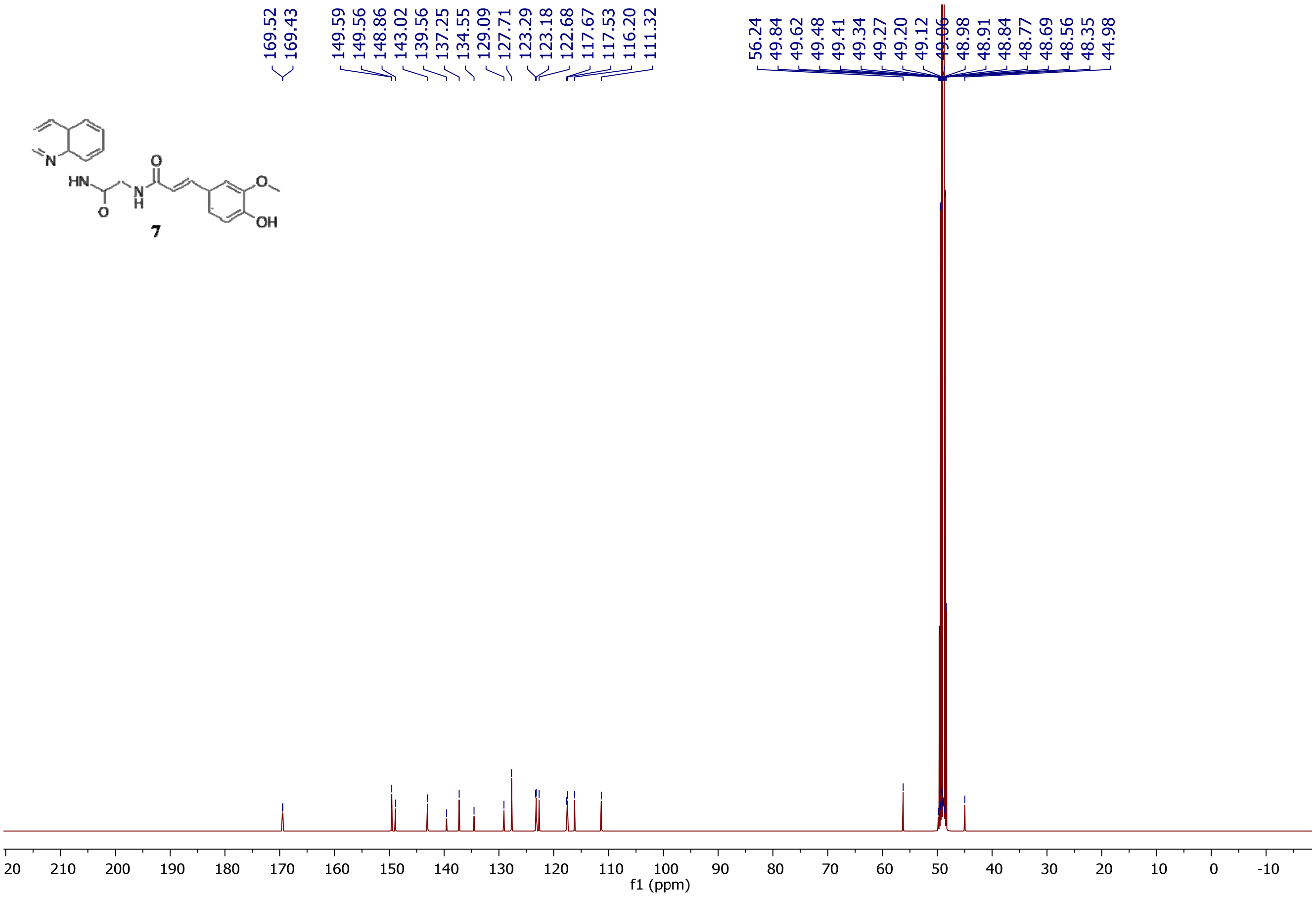

(E)-4-(3-(4-hydroxy-3-methoxyphenyl)acrylamido)-N-(quinolin-8-yl)butanamide **(8)**

^{1}H-NMR

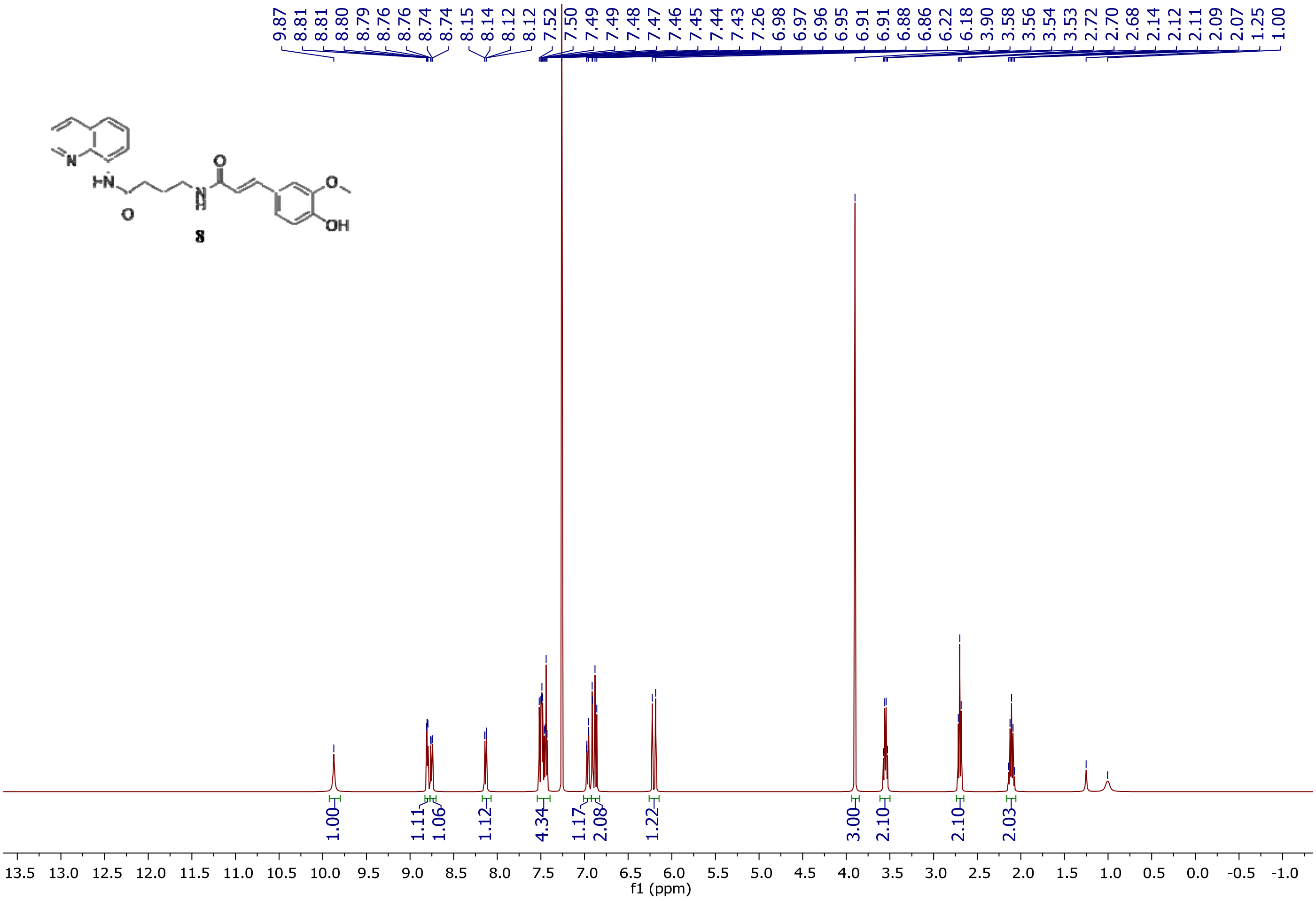
9.87 8.81 8.81 8.80 8.79 8.76 8.76 8.74 8.74 8.15 8.14 8.12 8.12 7.52 7.50 7.49 7.49 7.48 7.47 7.46 7.45 7.44 7.43 7.26 6.98 6.97 6.96 6.95 6.91 6.91 6.88 6.86 6.22 6.18 3.90 3.58 3.56 3.54 3.53 2.72 2.70 2.68 2.14 2.12 2.11 2.09 2.07 1.25 1.00
N
HN
O
N
H
O
O
OH
8
1.00 1.11 1.06 1.12 4.34 1.17 2.08 1.22 3.00 2.10 2.10 2.03
13.5 13.0 12.5 12.0 11.5 11.0 10.5 10.0 9.5 9.0 8.5 8.0 7.5 7.0 6.5 6.0 5.5 5.0 4.5 4.0 3.5 3.0 2.5 2.0 1.5 1.0 0.5 0.0 -0.5 -1.0
f1 (ppm)

^{13}C-NMR

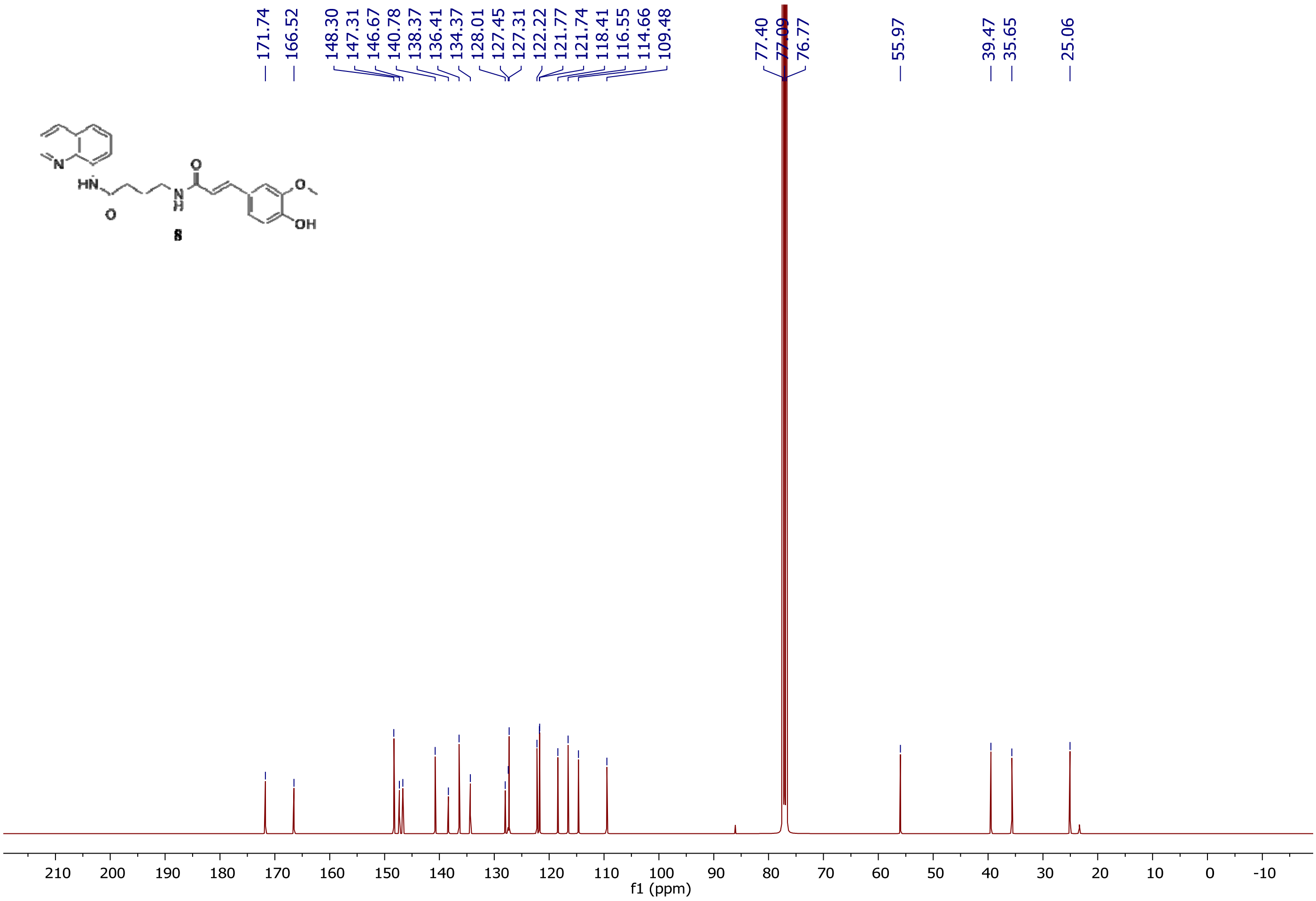

2. HPLC analysis

HPLC purity determination was performed on a Varian Pro Star 330PDA detector, a ternary HPLC pump Varian 9012 and a Rehodyne injector with 20 l loop. A RP column ThermosScientific TM Hypersil TM C18 ODS (5 m, 250 x 4.6 mmID) HPLC was used for all analysis (detection at 240 nm). Stock solutions of compounds 1-8 were prepared in a mixture of MeOH/ACN (2:3) and stored at 4°C. As the mobile phase, acetonitrile was used as eluent (A) and eluent (B) was water, with the elution gradient varying according to the method depicted in the table. HPLC analysis confirmed the ≥95% purity of all compounds 1-8.

Time (minutes)	Flow rate (mL/min)	%ACN (A)	%H_2O (B)
Initial	0.60	20	80
20 min	0.60	80	20
30 min	0.60	20	80

Compound 1

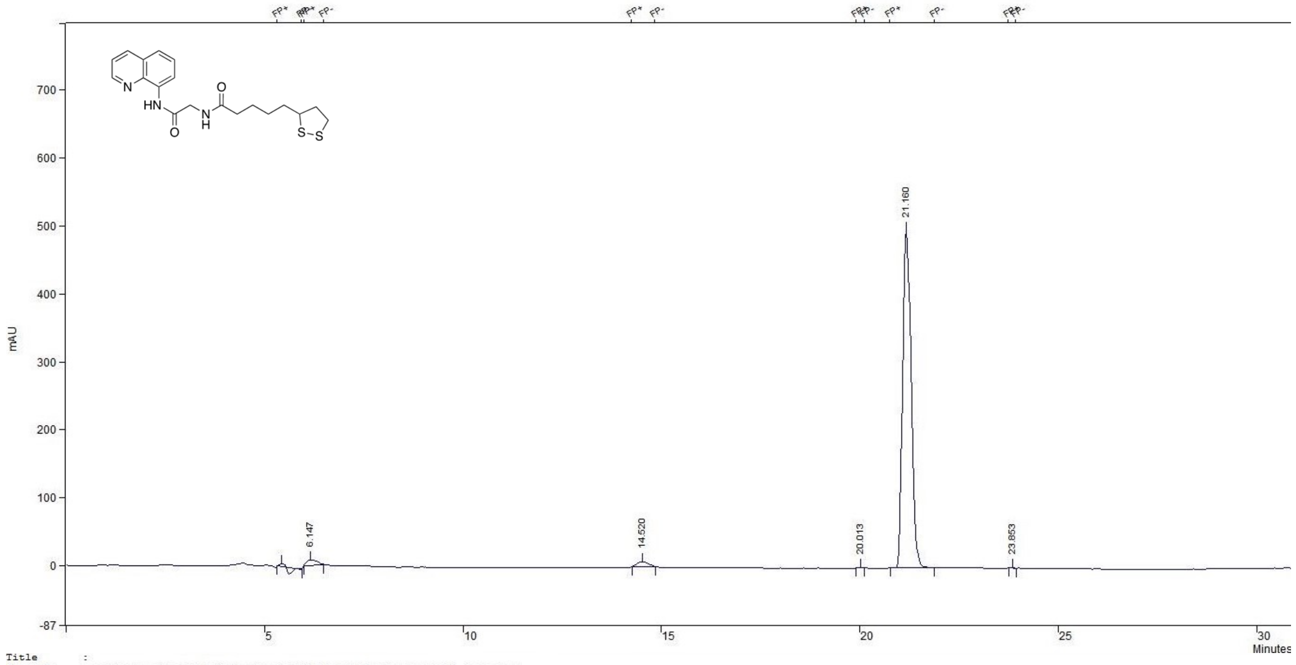


```
Title        :
Run File     : c:\documents and settings\varian\desktop\bacci\compound 1 h acn.run
Method File  : c:\star\data\bacci\si58 h acn 1-12 c-2.mth
Sample ID    : si58 h acn 1-12 c

Injection Date: 11/05/2022 12.53    Calculation Date: 11/05/2022 13.49

Operator    :                       Detector Type: 330 UV-Vis. PDA
Workstation: HPLC                   Bus Address  : 71
Instrument : Varian Star #1         Sample Rate  : 0.63 Hz
Channel    : 2 = 240.00 nm          Run Time     : 31.013 min

** LC Workstation Version 6.41 ** 01938-61c0-ea4-04b0 **

Run Mode          : Analysis
Peak Measurement: Peak Area
Calculation Type: Percent
```

Peak No.	Peak Name	Result ()	Ret. Time (min)	Time Offset (min)	Area (counts)	Sep. Code	Width 1/2 (sec)	Status Codes
1		2.0315	6.147	0.000	801499	BB	21.3	
2		1.8085	14.520	0.000	713510	BB	19.5	
3		0.0545	20.013	0.000	21515	BB	8.6	
4		96.0749	21.160	0.000	37905088	BB	14.2	
5		0.0306	23.853	0.000	12080	BB	6.1	
	Totals:	100.0000		0.000	39453692			

Compound 2

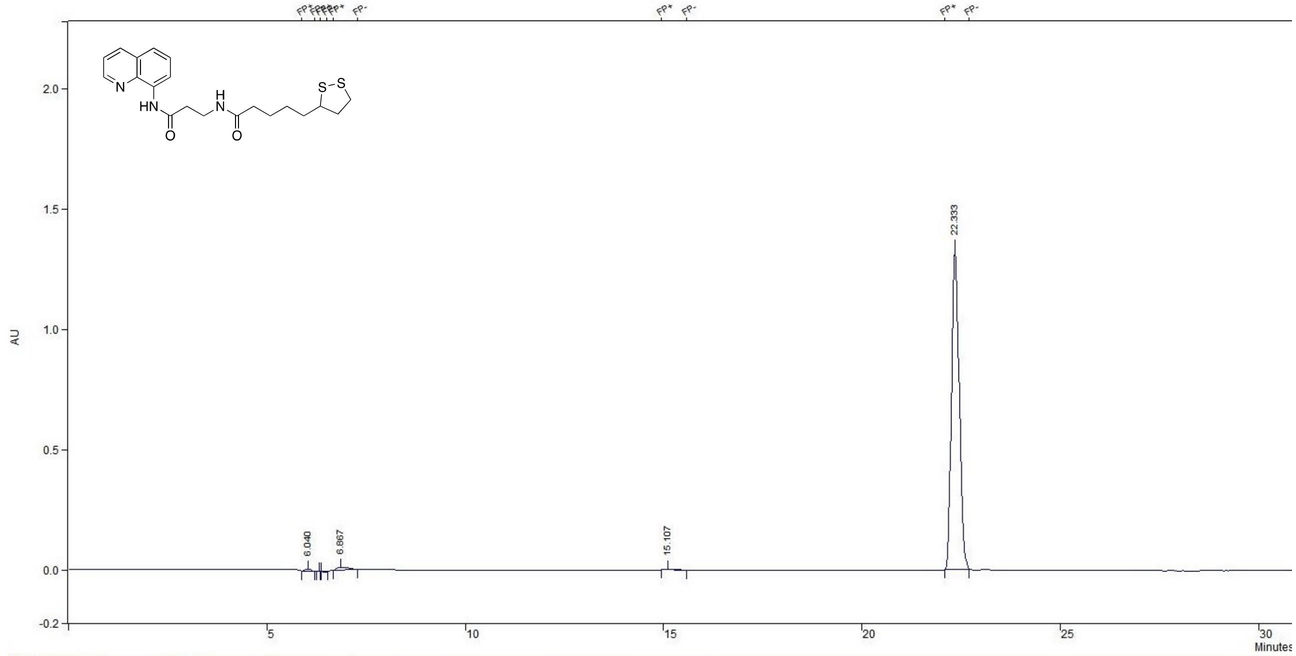

Title :
Run File : c:\documents and settings\varian\desktop\bacci\compound 2 h acn.run
Method File : c:\star\data\bacci\si 56 h acn 1-12 c-2.mth
Sample ID : SI 64 H ACN 1-12

Injection Date: 09/05/2022 16.52 Calculation Date: 09/05/2022 17.58

Operator :
Workstation: HPLC
Instrument : Varian Star #1
Channel : 2 = 240.00 nm

Detector Type: 330 UV-Vis. PDA
Bus Address : 71
Sample Rate : 0.63 Hz
Run Time : 31.013 min

** LC Workstation Version 6.41 ** 01938-61c0-ea4-04b0 **

Run Mode : Analysis
Peak Measurement: Peak Area
Calculation Type: Percent

Peak No.	Peak Name	Result ()	Ret. Time (min)	Time Offset (min)	Area (counts)	Sep. Code	Width 1/2 (sec)	Status Codes
1		0.5170	6.040	0.000	488785	BB	10.1	
2		1.1710	6.867	0.000	1107053	BB	23.3	
3		0.3042	15.107	0.000	287635	BB	13.2	
4		98.0078	22.333	0.000	92657848	BB	12.9	
	Totals:	100.0000		0.000	94541321			

Compound 3

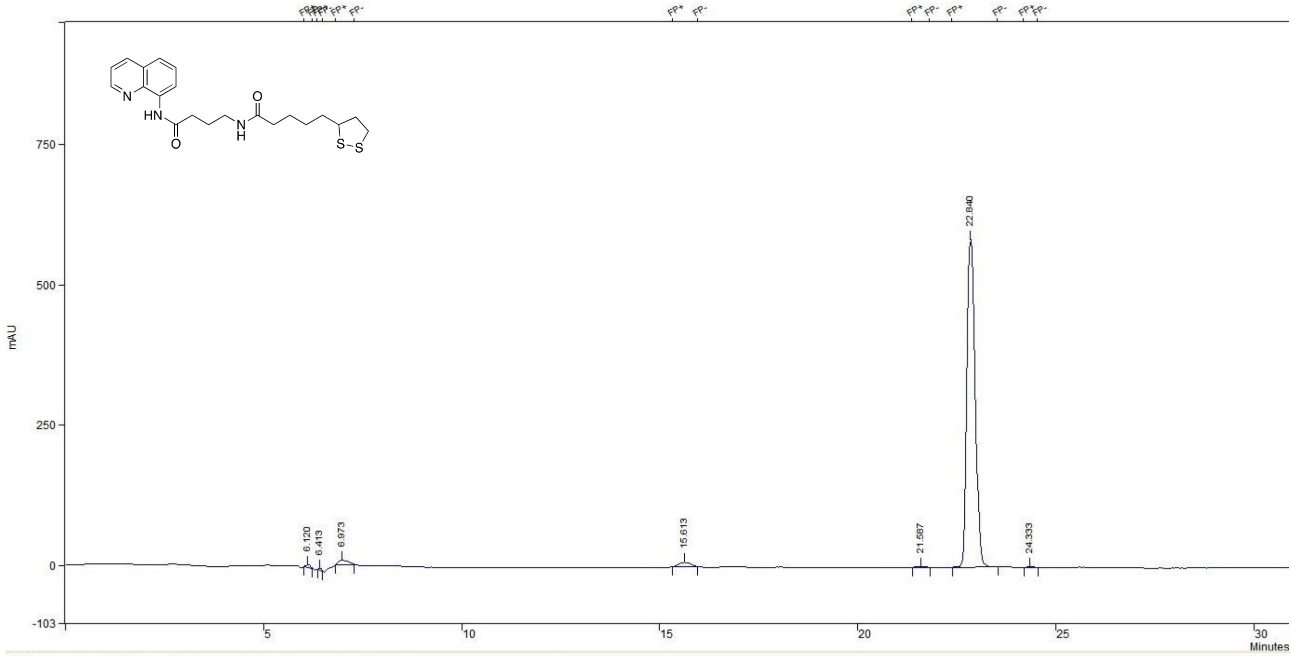


```
Title       :
Run File    : c:\documents and settings\varian\desktop\bacci\compound 3 h acn.run
Method File : c:\star\data\bacci\si 56 h acn 1-12 c-2.mth
Sample ID   : SI56 H ACN H 1-12 C

Injection Date: 09/05/2022 13.59    Calculation Date: 13/05/2022 10.54

Operator   :                        Detector Type: 330 UV-Vis. PDA
Workstation: HPLC                   Bus Address  : 71
Instrument : Varian Star #1         Sample Rate  : 0.63 Hz
Channel    : 2 = 240.00 nm          Run Time     : 31.013 min

** LC Workstation Version 6.41 ** 01938-61c0-ea4-04b0 **

Run Mode          : Analysis
Peak Measurement: Peak Area
Calculation Type: Percent
```

Peak No.	Peak Name	Result ()	Ret. Time (min)	Time Offset (min)	Area (counts)	Sep. Code	Width 1/2 (sec)	Status Codes
1		0.4992	6.120	0.000	226791	BB	7.3	
2		0.1659	6.413	0.000	75357	BB	3.4	
3		1.4702	6.973	0.000	667949	BB	18.6	
4		1.7996	15.613	0.000	817613	BB	21.3	
5		0.2441	21.587	0.000	110917	BB	15.8	
6		95.7174	22.840	0.000	43487596	BB	13.8	
7		0.1036	24.333	0.000	47080	BB	13.3	
	Totals:	100.0000		0.000	45433303			

Compound 4

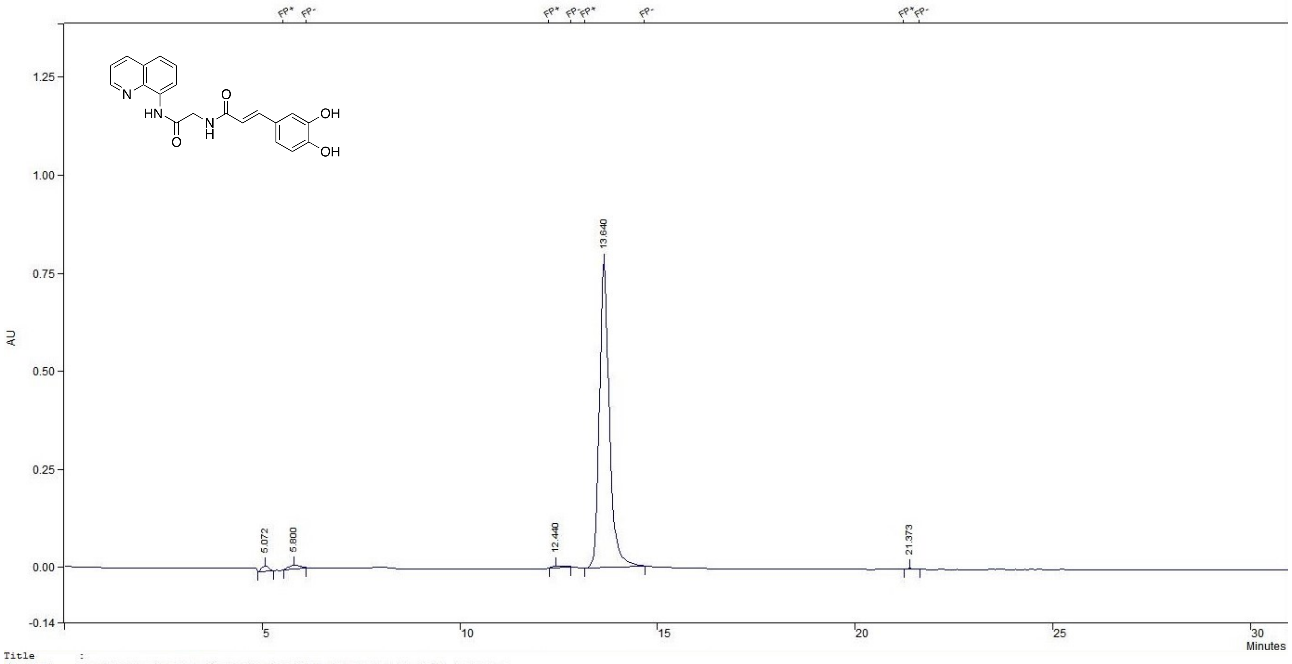


```
Title        :
Run File     : c:\documents and settings\varian\desktop\bacci\compound 4 h acn.run
Method File  : c:\star\data\bacci\si 27 h acn-2.mth
Sample ID    : SI 27 H ACN

Injection Date: 06/05/2022 10.18    Calculation Date: 11/05/2022 15.32

Operator   :                         Detector Type: 330 UV-Vis. PDA
Workstation: HPLC                    Bus Address  : 71
Instrument : Varian Star #1          Sample Rate  : 0.63 Hz
Channel    : 2 = 240.00 nm           Run Time     : 31.013 min

** LC Workstation Version 6.41 ** 01938-61c0-ea4-04b0 **

Run Mode         : Analysis
Peak Measurement: Peak Area
Calculation Type: Percent
```

Peak No.	Peak Name	Result ()	Ret. Time (min)	Time Offset (min)	Area (counts)	Sep. Code	Width 1/2 (sec)	Status Codes
1		1.0883	5.072	0.000	791303	BB	13.1	
2		1.2115	5.800	0.000	880839	BB	26.2	
3		0.5502	12.440	0.000	400043	BB	30.3	
4		97.0028	13.640	0.000	70528120	BB	15.2	
5		0.1471	21.373	0.000	106976	BB	10.4	
	Totals:	99.9999		0.000	72707281			

Compound 5

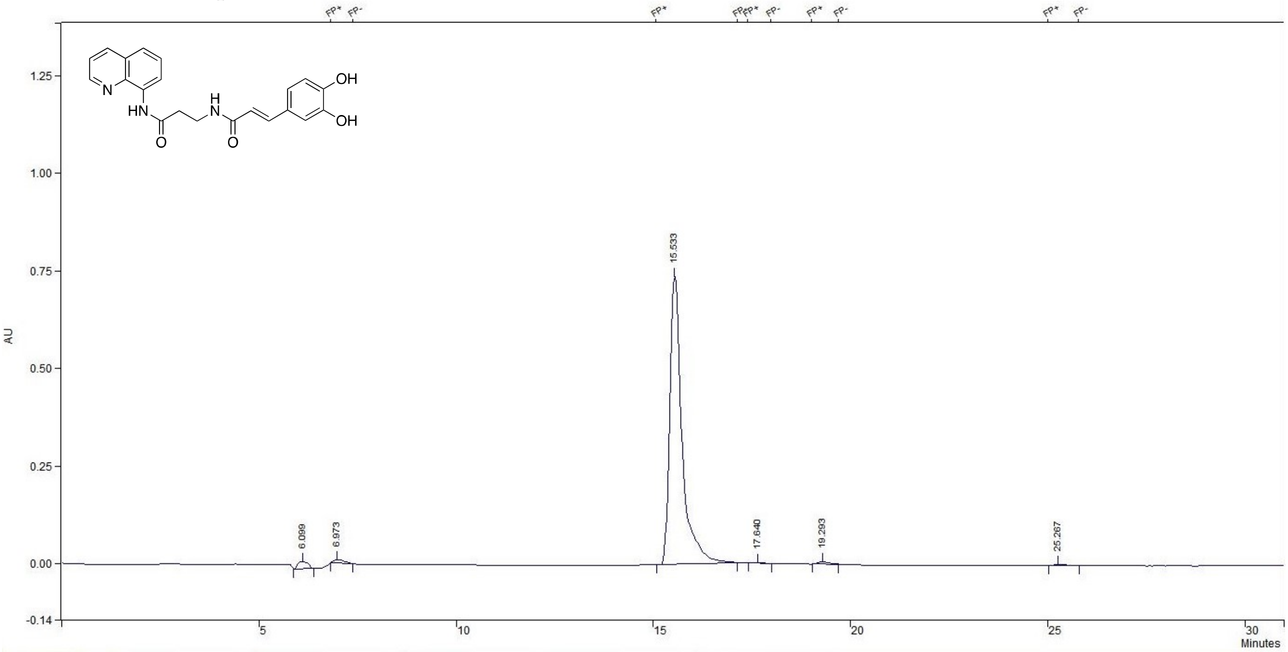

Title :
Run File : c:\documents and settings\varian\desktop\bacci\compound 5 h acn.run
Method File : c:\star\data\bacci\si 63 h acn 1-6-2.mth
Sample ID : SI 63 H ACN 1-6

Injection Date: 06/05/2022 15.08 Calculation Date: 13/05/2022 10.50

Operator : Detector Type: 330 UV-Vis. PDA
Workstation: HPLC Bus Address : 71
Instrument : Varian Star #1 Sample Rate : 0.63 Hz
Channel : 2 = 240.00 nm Run Time : 31.013 min

** LC Workstation Version 6.41 ** 01938-61c0-ea4-04b0 **

Run Mode : Analysis
Peak Measurement: Peak Area
Calculation Type: Percent

Peak No.	Peak Name	Result ()	Ret. Time (min)	Time Offset (min)	Area (counts)	Sep. Code	Width 1/2 (sec)	Status Codes
1		1.9285	6.099	0.000	1654871	BB	19.0	
2		0.9576	6.973	0.000	821741	BB	17.6	
3		95.9189	15.533	0.000	82310720	BB	17.8	
4		0.2148	17.640	0.000	184284	BB	12.7	
5		0.6672	19.293	0.000	572517	BB	17.8	
6		0.3131	25.267	0.000	268664	BB	20.5	
	Totals:	100.0001		0.000	85812797			

Compound 6

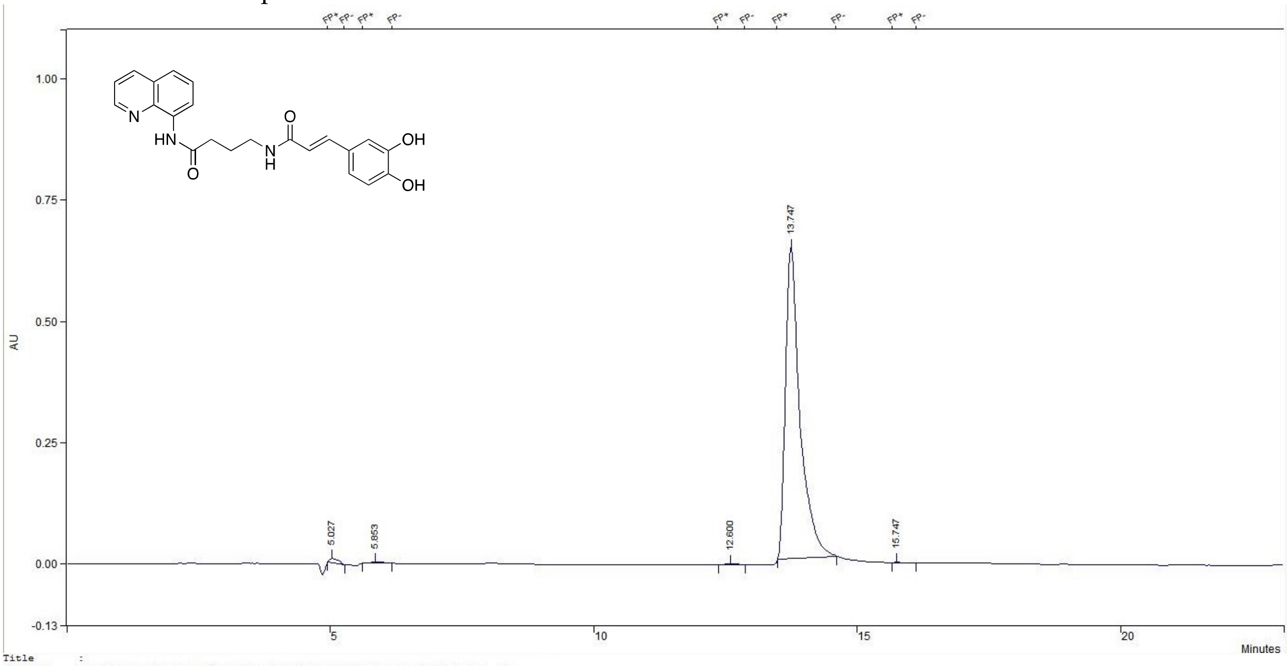

Title :
Run File : c:\documents and settings\varian\desktop\bacci\compound 6 h acn .run
Method File : c:\star\data\bacci\si 24 h acn -2.mth
Sample ID : sSI 24 H ACN

Injection Date: 05/05/2022 16.38 Calculation Date: 13/05/2022 10.49

Operator : Detector Type: 330 UV-Vis. PDA
Workstation: HPLC Bus Address : 71
Instrument : Varian Star #1 Sample Rate : 0.63 Hz
Channel : 2 = 240.00 nm Run Time : 23.120 min

** LC Workstation Version 6.41 ** 01938-61c0-ea4-04b0 **

Run Mode : Analysis
Peak Measurement: Peak Area
Calculation Type: Percent

Peak No.	Peak Name	Result ()	Ret. Time (min)	Time Offset (min)	Area (counts)	Sep. Code	Width 1/2 (sec)	Status Codes
1		0.7476	5.027	0.000	482938	BB	9.2	
2		0.4164	5.853	0.000	268990	BB	15.7	
3		0.1795	12.600	0.000	115946	BB	19.8	
4		98.5946	13.747	0.000	63687904	BB	16.1	
5		0.0619	15.747	0.000	39989	BB	4.4	
	Totals:	100.0000		0.000	64595767			

Compound 7

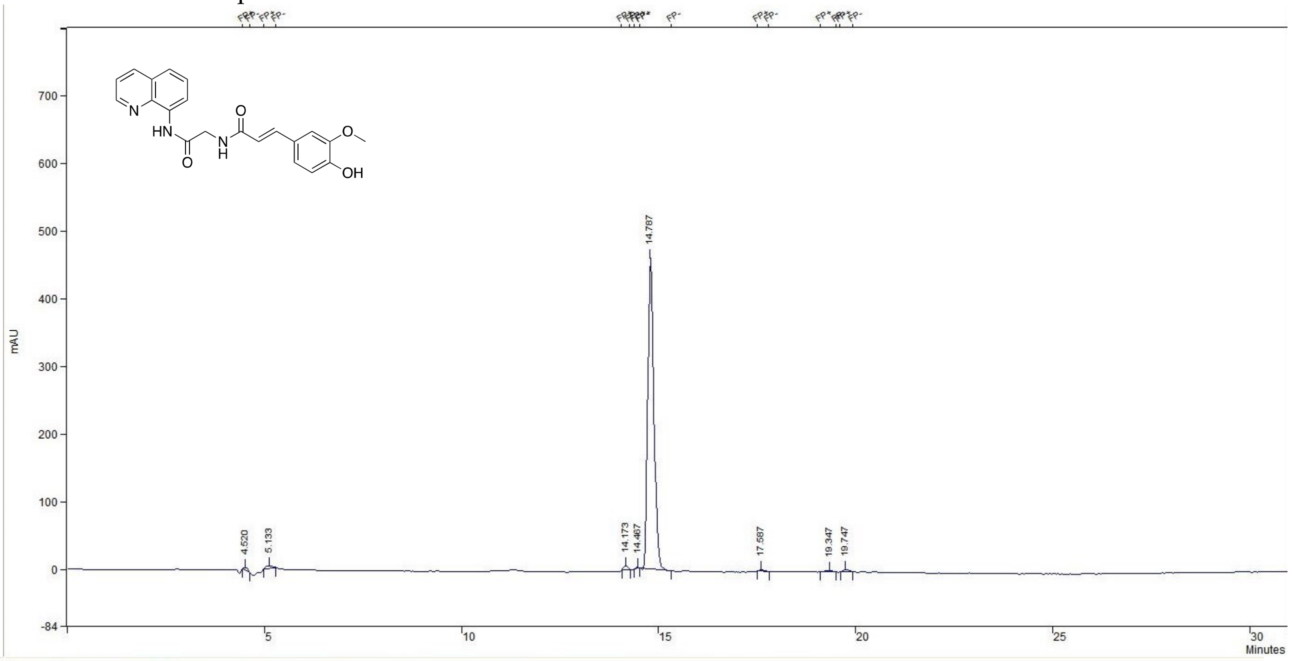


```
Title       :
Run File    : c:\documents and settings\varian\desktop\bacci\compound 7 h acn.run
Method File : c:\star\data\bacci\si 37 h acn 1-6 at2-2.mth
Sample ID   : si 37 h acn 1-6 at2

Injection Date: 12/05/2022 16.58    Calculation Date: 12/05/2022 18.53

Operator   :                        Detector Type: 330 UV-Vis. PDA
Workstation: HPLC                   Bus Address  : 71
Instrument : Varian Star #1         Sample Rate  : 0.63 Hz
Channel    : 2 = 240.00 nm          Run Time     : 31.013 min

** LC Workstation Version 6.41 ** 01938-61c0-ea4-04b0 **

Run Mode          : Analysis
Peak Measurement: Peak Area
Calculation Type: Percent
```

Peak No.	Peak Name	Result ()	Ret. Time (min)	Time Offset (min)	Area (counts)	Sep. Code	Width 1/2 (sec)	Status Codes
1		0.6667	4.520	0.000	175246	BB	6.1	
2		0.8573	5.133	0.000	225365	BB	15.9	
3		0.6451	14.173	0.000	169566	BB	7.8	
4		0.1833	14.467	0.000	48174	BB	5.8	
5		96.1359	14.787	0.000	25271500	BB	9.9	
6		0.3115	17.587	0.000	81891	BB	7.9	
7		0.4977	19.347	0.000	130838	BB	14.0	
8		0.7025	19.747	0.000	184674	BB	10.3	
	Totals:	100.0000		0.000	26287254			

Compound 8

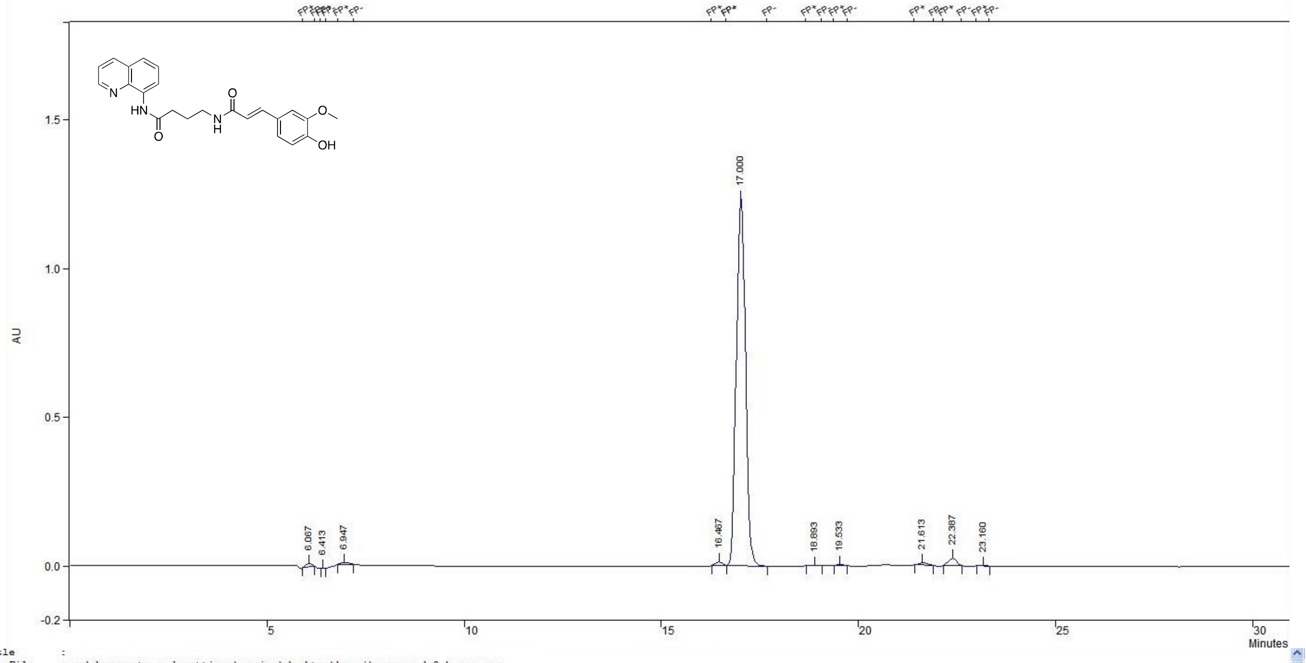


```
Title        :
Run File     : c:\documents and settings\varian\desktop\bacci\compound 8 h acn.run
Method File  : c:\star\data\bacci\si 38 h acn 1-6-2.mth
Sample ID    : SI 38 H ACN 1-6

Injection Date: 06/05/2022 15.48    Calculation Date: 13/05/2022 10.53

Operator    :                       Detector Type: 330 UV-Vis. PDA
Workstation: HPLC                   Bus Address  : 71
Instrument : Varian Star #1         Sample Rate  : 0.63 Hz
Channel     : 2 = 240.00 nm         Run Time     : 31.013 min

** LC Workstation Version 6.41 ** 01938-61c0-ea4-04b0 **

Run Mode          : Analysis
Peak Measurement: Peak Area
Calculation Type: Percent
```

Peak No.	Peak Name	Result ()	Ret. Time (min)	Time Offset (min)	Area (counts)	Sep. Code	Width 1/2 (sec)	Status Codes
1		0.5590	6.067	0.000	601926	BB	14.5	
2		0.0210	6.413	0.000	22560	BB	2.8	
3		0.3947	6.947	0.000	424989	BB	16.5	
4		0.6452	16.467	0.000	694736	BB	12.3	
5		95.6712	17.000	0.000	103013872	BB	15.4	
6		0.1053	18.893	0.000	113403	BB	17.7	
7		0.2048	19.533	0.000	220512	BB	11.1	
8		0.5740	21.613	0.000	618067	BB	14.3	
9		1.7160	22.387	0.000	1847678	BB	14.8	
10		0.1088	23.160	0.000	117109	BB	9.9	
	Totals:	100.0000		0.000	107674852			

3. Copper chelating study

Standard solutions of compounds 1-8 were prepared in 100% DMSO and diluted in absolute ethanol to reach the concentration of 20 μM (DMSO final concentration < 0.1%). A 4X stock solution of $CuCl_2$ was prepared at concentration of 800 μM in absolute ethanol. 100 μL of this solution was transfected in a 96-well plate and diluted to obtain progressive final concentrations of 100, 50, 25, 12.5, 6.25, 3.125, 1.56, 0 μM. Then, 100 μL of each compound solutions were added in every well obtaining a final concentration of compounds of 10 μM (duplicated). After 30 minutes of incubation in a dark room, the absorption spectrum was recorded at room temperature with UV-vis spectrophotometer PerkinElmer EnSpire 2300. Absorption spectra were collected detecting values at different wavelengths (230-500 nm) and normalized with control (ethanol solution) and then analyzed the absorbance values in function of wavelength using SpectraGryph 1.2.

4. DPPH assay

In this method, the scavenging activity of the colored radical by synthetized compounds is followed by spectrophotometry. Standard solutions of compounds **1-8** were prepared in 100% DMSO and diluted in absolute methanol to reach the concentration of 400 μM (DMSO final concentration < 0.1%). 75 μL of each compound solution were properly diluted to 200 and 100 μM and then added with 75 μL of 1mM methanolic solution of DPPH (final DPPH concentration 500 μM, final concentrations of compounds 200, 100 and 50 μM, duplicated). The well was incubated in a dark room, at room temperature for 45 minutes and the absorbance values was measured at 531 nm with PerkinElmer Enspire 2300 multiplate reader. Instead, for detect IC_{50} of selected compounds, in a 96-well plate 300 μM methanolic solutions of compounds (75 μL) were properly diluted to 150, 75, 37.5, 18.8, 9.4, 4.7 and 0 μM (duplicated). Then, in each well were added 75 μL of a 1 mM methanolic solutions of DPPH (final well concentration 500 nM). The well was incubated in a dark room, at room temperature for 45 minutes and the absorbance values was measured at 531 nm with PerkinElmer Enspire 2300 multiplate reader. All the values were then mediated, normalized with control (only methanol) and the scavenging activity percentage calculated with the formula:

$$scavenging\ capacity\,\% = 100 * \frac{Abs_{DPPH} - Abs_{compound}}{Abs_{DPPH}}$$

5. Cell viability

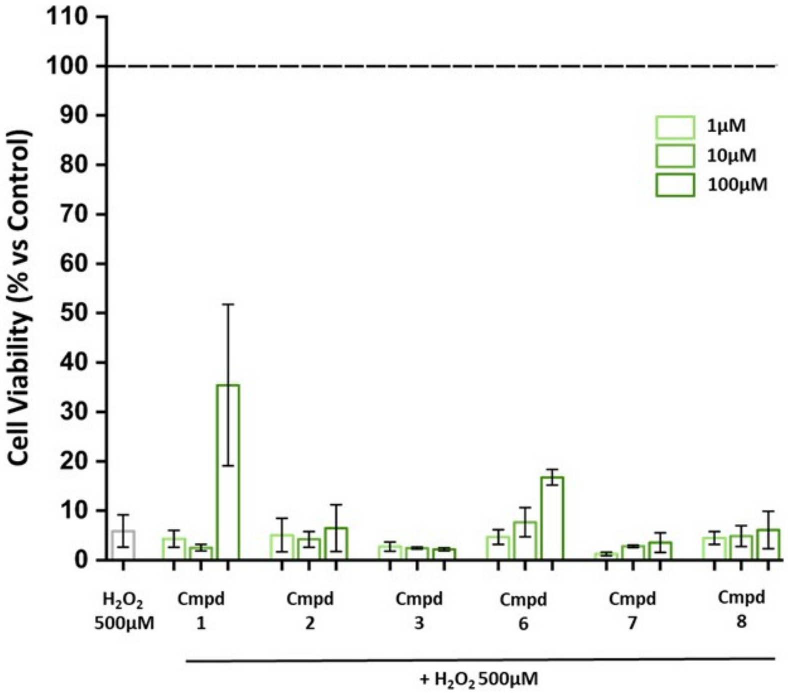

Figure S1. Cell viability was analysed by CellTiter 96 Aqueous – One solution Reagent. Cells were pre-treated for 24 h with compounds at various concentration (1-10-100μM) and then exposed for 3 h with H_2O_2 500μM. The dashed line indicates the reference value of Ctrl: control group - no compounds or H_2O_2 exposure. Values in the graph indicate % viability as the mean ± SE obtained from a n=3 of independent experiments
